# Nucleophosmin 1 cooperates with the methyltransferase DOT1L to preserve peri-nucleolar heterochromatin organization by regulating H3K27me3 levels and DNA repeats expression

**DOI:** 10.1186/s13072-023-00511-9

**Published:** 2023-09-28

**Authors:** Annalisa Izzo, Ipek Akol, Alejandro Villarreal, Shannon Lebel, Marta Garcia-Miralles, Arquimedes Cheffer, Patrick Bovio, Stefanie Heidrich, Tanja Vogel

**Affiliations:** 1 Department of Molecular Embryology, Institute of Anatomy and Cell Biology, Department of Molecular Embryology, Medical Faculty, Albert-Ludwigs-University Freiburg, 79104 Freiburg, Germany; 2 Laboratorio de Neuropatología Molecular, Facultad de Medicina, Instituto de Biología Celular y Neurociencia “Prof. E. De Robertis” UBA-CONICET, Universidad de Buenos Aires, 1121 Buenos Aires, Argentina; 3 Faculty of Biology, Albert-Ludwigs-University of Freiburg, 79104 Freiburg, Germany; 4 Center for Basics in NeuroModulation (NeuroModul Basics), Medical Faculty, Albert-Ludwigs-University Freiburg, 79104 Freiburg, Germany

**Keywords:** DOT1L, NPM1, Histone methylation, Peri-nucleolar heterochromatin, Transcription, Epigenetics

## Abstract

**Background:**

NPM1 is a phosphoprotein highly abundant in the nucleolus. However, additional nuclear functions have been attributed to NPM1, probably through interaction with other nuclear factors. DOT1L is one interaction partner of NPM1 that catalyzes methylation of histone H3 at lysine 79 (H3K79). DOT1L, playing functional roles in several biological processes, is known for its capability to organize and regulate chromatin. For example, DOT1L modulates DNA repeats expression within peri-nucleolar heterochromatin. NPM1 also affects peri-nucleolar heterochromatin spatial organization. However, it is unclear as of yet whether NPM1 and DOT1L functionally synergize to preserve nucleoli organization and genome stability, and generally, which molecular mechanisms would be involved.

**Results:**

We characterized the nuclear function of NPM1 on peri-nucleolar heterochromatin organization. We show that (i) monomeric NPM1 interacts preferentially with DOT1L in the nucleus; (ii) NPM1 acts in concert with DOT1L to maintain each other’s protein homeostasis; (iii) NPM1 depletion results in H3K79me2 upregulation and differential enrichment at chromatin binding genes including *Ezh2*; (iv) NPM1 and DOT1L modulate DNA repeats expression and peri-nucleolar heterochromatin organization via epigenetic mechanisms dependent on H3K27me3.

**Conclusions:**

Our findings give insights into molecular mechanisms employed by NPM1 and DOT1L to regulate heterochromatin activity and structural organization around the nucleoli and shed light on one aspect of the complex role of both proteins in chromatin dynamics.

**Supplementary Information:**

The online version contains supplementary material available at 10.1186/s13072-023-00511-9.

## Background

NPM1 is a highly abundant phospho-protein in the granular region of the nucleolus, it contains a nuclear localization signal (NLS) and can shuttle between the nucleus and cytosol (Szebeni, Herrera and Olson, [8, 61]. It occurs in mono- or oligomeric forms [16], and the equilibrium between these two states is crucial for the localization and function of NPM1 [4] [50]. Phosphorylated, monomeric NPM1 localizes predominantly in the nucleus at sites of DNA damage [34], whereas nucleolar NPM1 seems mainly oligomeric [45]. NPM1 can also act as RNA binding protein and the RNA binding activity affects its intracellular localization [56]. Specifically, RNA binding favors oligomerization of NPM1 and its localization in the nucleolus. Monomeric NPM1 instead can be found on chromatin in the nucleoplasm [45, 46]. Apart from these findings it is not well understood as of yet, how the dynamics of NPM1 state conversion occur [56].

NPM1 has been widely characterized for its oncogenic functions: it is often overexpressed in solid tumors [32] and it is the most frequently mutated gene in acute myeloid leukemia (AML) [65]. The majority of NPM1 mutations occur in the C-terminal DNA-binding domain and result in its aberrant cytosolic localization (NPM1c + AML) [18]. Recently it has been shown that NPM1c is also recruited to chromatin to maintain active transcription of target genes driving leukemia [68]. Additional biological functions have been attributed to NPM1, including cell proliferation [34, 51], ribosome biogenesis [30, 39, 57], and chromatin remodeling through its function as histone chaperone [17]. Moreover, the knockdown of NPM1 in neural stem cells reduces proliferation and increases apoptosis without affecting neuronal differentiation [52]. Notably, not only the loss of NPM1 but also its ectopic expression in differentiated neurons leads to reduced neuronal survival, possibly due to its preferred occurrence in the oligomeric state [50]. This finding suggests that NPM1 has an important, yet mainly unknown, role in cells of the neural lineage. The interaction of NPM1 with different cofactors underlies this plethora of biological roles.

Among others, the Disruptor of telomeric silencing-like 1 (DOT1L) is a prominent interaction partner of NPM1 [49]. DOT1L is a methyltransferase that mono-, di- and tri-methylates histone H3 at lysine 79 (H3K79) [19, 67]. DOT1L regulates gene transcription at multiple levels, but the specific role of DOT1L in transcriptional regulation is still under debate, as it seemingly depends on experimental conditions and the respective model system used. For example, the histone mark conferred by DOT1L, i.e. H3K79 methylation (me), has been initially associated with active genes [12, 22], however it also marks repressed regions [10, 21] [76]. Further, DOT1L might either regulate productive transcriptional elongation and/or transcriptional initiation [11] (A. [70, 71]. Regarding posttranscriptional events, H3K79me2 seems to play a functional and regulatory role in alternative splicing, at least in cancer cells [38].

In addition to controlling several aspects of transcriptional regulation, DOT1L participates in different cellular processes such as cell proliferation [33], embryonic stem cell (ESC) differentiation [5], DNA repair [31] and somatic cell reprograming [69]. During development, DOT1L controls differentiation and survival of neural stem cells. In this context, inhibition of DOT1L induces death of cultured cortical neurons by activating ER stress-related transcriptional programs [54]. Further, DOT1L maintains neural progenitor proliferation, primes cortical neuron precursor cells for layer sub-type specification during brain development and prevents premature neuronal differentiation [2, 10, 20, 21].

Like NPM1, DOT1L has been implicated in the pathogenesis of AML, bearing the MLL-AF9 (MLLT3)/ AF10 (MLLT10) fusion proteins. Here, through its association with the transcription factors AF9 and AF10, DOT1L is recruited to MLL target genes, which results in their aberrant methylation at H3K79 and the establishment of the malignant phenotype [15, 47].

Despite an increasing, but so far limited understanding of individual DOT1L and NPM1 functions, our knowledge of the implications of their cooperation and crosstalk is even less complete. A few insights come from studies in NPM1c + AML [25, 27], in which combined pharmacological inhibition of DOT1L activity and menin-MLL1 interaction significantly reduced the expression of oncogenes [35]. Moreover, a human NPM1c + AML cell line contains increased levels of H3K79me2 compared to healthy donor cells [77].

Other functional associations between NPM1 and DOT1L can be anticipated. On one hand, NPM1 expression maintains the nucleolar shape and peri-nucleolar heterochromatin organization (Holmberg Olausson, Nistér and Lindström, [28]). This chromatin-shaping function of NPM1 might be mediated by its interaction with the DNA binding factor CTCF (CCCTC-Binding Factor). NPM1 contributes to the CTCF-mediated transcriptional insulation by tethering CTCF binding sites to the nucleolar periphery and inducing heterochromatin silencing [75]. On the other hand, DOT1L enzymatic activity is required for the burst of transcription of repetitive DNA elements at peri-centromeric heterochromatin, which is necessary for their proper silencing and maintenance of genomic stability [42]. Together, these findings suggest that NPM1 and DOT1L have an important role in peri-nucleolar heterochromatin organization and establishment/maintenance of nucleoli structure. However, the nature of an underlying molecular mechanism that links NPM1 and DOT1L function(s), and which could be either conferred by direct protein interaction or by convergence in similar pathways/regulative networks is largely unknown.

In this work, we addressed a potential synergy between mouse NPM1 and DOT1L, with a specific focus on heterochromatin organization around nucleoli in the neuroblastoma N2a cell line. We show that monomeric NPM1 interacts with DOT1L in the nucleus at the chromatin level. Extending on individual, yet synergistic functions, we identified a novel regulatory feedback loop that involves both NPM1 and DOT1L. NPM1 and DOT1L modulate the epigenetic landscape and contribute to the maintenance of DNA repeats silencing within peri-nucleolar heterochromatin, whereas “DNA repeats” refer to both tandem repeats (DNA satellites) and interspersed repeats (IAP, LINE, MERVL, ETN). Mechanistically, NPM1 and DOT1L contribute to the organization of heterochromatin around nucleoli through a crosstalk with H3K27me3. This study of NPM1 and DOT1L cooperation is of crucial relevance to understand the multifaceted ways of action of both proteins. Our finding of a synergistic role of NPM1 and DOT1L in maintaining heterochromatin functions around the nucleoli, and consequently in genome stability, provides a possible molecular mechanisms to explain malignant phenotypes of some cancer cells (for example NPM1c + types of leukemia), and thus has also important clinical implications.

## Results

### Monomeric NPM1 interacts with DOT1L on chromatin

**Fig. 1:**
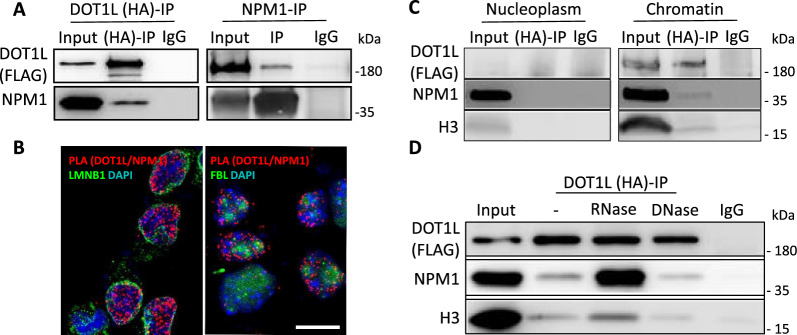
DOT1L interacts with monomeric NPM1 on chromatin. **A** DOT1L (DOT1L-FLAG-HA) was overexpressed in N2a cells and immunoprecipitated using HA antibody (left panel) or NPM1 antibody (right panel). IgG antibody was used as control. For DOT1L immune-detection FLAG antibody was used. **B** Proximity ligation assay (in situ PLA) to visualize in vivo NPM1/DOT1L interaction (red signal) when both proteins are overexpressed in N2a cells. LMNB1 (LAMINB1) and FBL (FIBRILLARIN) were used as controls to mark the nuclear membrane and the nucleoli respectively. DOT1L/NPM1 interaction occurred inside the nucleus (LMNB1) and outside the nucleolus (FBL). (Scale bar 10 µm). **C** Immunoblot analysis of DOT1L/NPM1 co-IP as in A) from nucleoplasm or chromatin of N2a cell fractions. Total histone 3 (H3) was used to control the purity of the cell fractionation. For DOT1L immune-detection FLAG antibody was used. **D** Immunoblot analysis of DOT1L/NPM1 interaction in N2a cell extract as in A) in the presence of DNase or RNase, respectively

Previous data showed co-immunoprecipitation (co-IP) of NPM1 with the methyltransferase DOT1L in human HEK293T cells [49], but further characterization of this protein complex was not yet addressed. As a starting point to investigate NPM1/DOT1L synergy in mouse cells, we used reciprocal co-IP followed by immunoblot analysis and detected endogenous NPM1 interacting with DOT1L in neuroblastoma N2a cells (Additional file 1: Fig. S1A). However, upon NPM1 immunoprecipitation the DOT1L antibody failed to recognize a band corresponding to endogenous DOT1L, probably due to the low sensitivity of this antibody for sub-stoichiometric amounts of DOT1L (Additional file 1: Fig. S1A). To overcome this technical problem, we overexpressed DOT1L-HA-FLAG in N2a cells and performed co-IP using antibodies against HA. Immunoblot revealed a positive band for NPM1 and DOT1L, indicating that both proteins were part of the same complex in these mouse cells (Fig. 1A). We confirmed NPM1-DOT1L interaction using a Proximity Ligation Assay (PLA) in N2a cells. Interestingly, the NPM1/DOT1L complex localized exclusively to the nucleus marked by LMNB1 (LAMINB1) but was excluded from the FBL (FIBRILLARIN) marked nucleolus, a nuclear compartment where NPM1 is normally highly enriched (Fig. 1B and Additional file 1: Fig. S1B). We further characterized the interaction of NPM1 and DOT1L in the nuclear compartments. Co-IPs from nucleoplasm and chromatin cell extracts revealed that NPM1/DOT1L interaction occurs at the chromatin level, but not in the nucleoplasm (Fig. 1C). Therefore, we concluded that NPM1 and DOT1L can be part of the same complex, located on chromatin around the nucleolus.

The intracellular localization of NPM1 is regulated by its RNA binding activity and oligomerization [56]. RNA binding favors the oligomerization of NPM1 and thereby localization in the nucleolus, whereas monomeric NPM1 localizes on chromatin in the nucleoplasm [45, 46]. To test for a DNA- or RNA-dependent NPM1/DOT1L interaction, we treated N2a cell lysates either with RNase or DNase. We observed a much stronger interaction in the presence of RNase compared to the non-treated control condition (Fig. 1D). DNase treatment, in turn, did not affect the binding of NPM1 with DOT1L. Therefore, we hypothesized that DOT1L interacts with monomeric NPM1, and that RNase treatment increases this interaction by shifting the equilibrium from the oligomeric towards the monomeric status. In support, co-IP in the presence of EGS (ethylene glycol bis(succinimidyl succinate)), a potent cross-linker of protein–protein interactions, revealed the presence of both oligomeric and monomeric NPM1 in the input, the latter of which indeed interacted preferentially with DOT1L (Additional file 1: Fig. S1C). These results showed that in mouse N2a cells monomeric NPM1 interacts with DOT1L and that this interaction is confined to the chromatin. As both NPM1 and DOT1L function at the chromatin level, our further experimental attempts aimed to reveal the synergistic effects of both partners (but not necessarily of the complex) on chromatin organization.

### Loss of NPM1 increases global H3K79me2 levels through upregulation of DOT1L

To test whether NPM1 would have regulatory effects on DOT1L activity impacting H3K79 methylation similar to human cells [77], we reduced NPM1 levels by transfecting mouse N2a cells with a shRNA construct (KD). In our experiments we could reach, at the protein level, a maximum of 75% (Additional file 1: Fig. S4E) in knockdown efficiency. Considering the key role of NPM1 in several vital biological functions (e.g. ribosome biogenesis, centrosome maintenance, [52]), it is presumable that perseverance of limiting amounts of NPM1 is critical for cell survival. We first characterized the effect of NPM1 KD on a cellular level, and observed three days after transfection that cells with NPM1 KD had a roundish morphology and consumed less culture medium than control cells (CTR) (Additional file 1: Fig. S2A). In addition, only a fraction of NPM1 KD cells showed expression of activated CASPASE 3 (aCASP3; Additional file 1: Fig. S2B, C), a marker of apoptosis, or exhibited nuclear fragmentation as revealed by DAPI staining (Additional file 1: Fig. S2B-C). We concluded that N2a cells tolerate NPM1 KD to an extent that allowed characterizing synergistic functions with DOT1L in further detail.

**Fig. 2:**
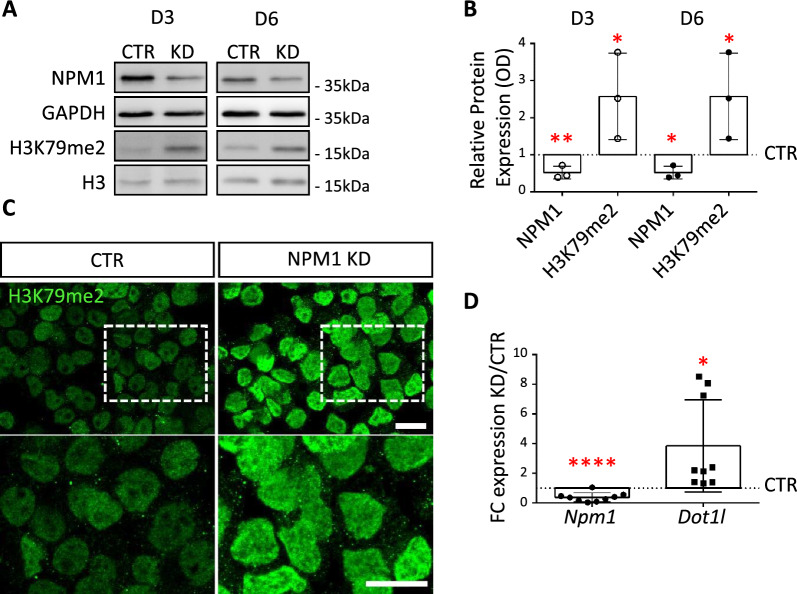
NPM1 KD increases H3K79me2 via upregulation of *Dot1l*. **A** Representative immunoblot analysis showing an increase in H3K79me2 levels after 3 or 6 days (3D, 6D) of NPM1 KD in N2a cells. Three biological replicates were used (n = 3). **B** Quantification of immunoblot signal intensities shown in A were conducted using Fiji (ImageJ) software. NPM1 and H3K79me2 levels were normalized to the levels of GAPDH or total H3, respectively. Secondly, the normalized value of each treatment was transformed as a fraction of its control which acquired the value of 1 (CTR = 1) and is shown with a dashed line on the graphs. Error bars represent S.D. Statistical analysis was performed using one sample t-test *p < 0.05, **p < 0.01 (n = 3). **C** Confocal immunofluorescence showing an example of H3K79me2 staining after 3 days of shNPM1 transfection (NPM1 KD) in N2a cells. (Scale bars 10 µm). **D** Individual data points plot showing the fold change (NPM1 KD/CTR) expression of the *Npm1* and *Dot1l* genes calculated after RT-qPCR upon 3 days of NPM1 KD in N2a cells. *Gapdh* was used as reference gene for normalization. Statistical analysis was performed on n = 9 biological replicates using an unpaired two tailed (*Dot1l*) and one tailed (*Npm1*) t-test; *p < 0.05, **p < 0.01. Error bars represent S.D

We next quantified H3K79me2 levels at three and six days after NPM1 KD by immunoblot analysis and observed significantly increased levels of H3K79me2 compared to cells transfected with a control shRNA (Fig. 2A, B). We confirmed increased levels of H3K79me2 by confocal microscopy imaging (Fig. 2C). H3K79me2 levels can fluctuate during the cell cycle, whereby highest levels were observed around the G2/M phase in both yeast and mammalian cells [58], (Stulemeijer et al., [19, 60]. To investigate if changes in H3K79me2 upon NPM1 KD depended on the cell cycle, we profiled the cell cycle of N2a cells using flow cytometry. This analysis showed that upon NPM1 KD cells progressed through the cell cycle similarly to the control cells. We did not observe significant differences in the distribution of the cells in the different phases of the cell cycle (Additional file 1: Fig. S2D). We concluded that increased levels of H3K79me2 upon NPM1 KD were not due to a specific block of the cells in G2/M. We thus hypothesized that changes in the expression of *Dot1l* accounted for the increased levels of H3K79me2 upon NPM1 KD. By employing RT-qPCR we identified an increased expression of *Dot1l* in the NPM1 KD condition (Fig. 2D), which was in accordance with the global increase in H3K79me2 levels upon reduced expression of NPM1.

Altogether, this indicates that the reduction of NPM1 expression in N2a cells increased H3K79me2 levels most likely due to increased expression of *Dot1l*.

### Reduction of NPM1 alters H3K79me2 at chromatin modifying genes

**Fig. 3:**
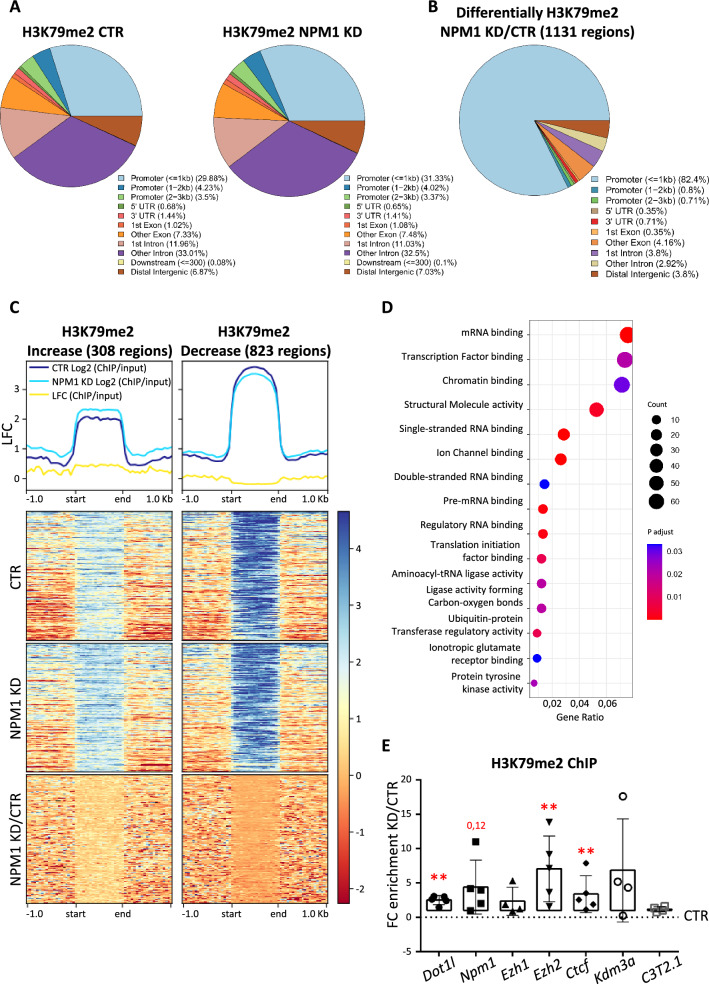
H3K79me2 is enriched at chromatin binding genes upon loss of NPM1. **A** Pie charts depicting the genomic distribution of H3K79me2 peaks in control (CTR, left), and after 3 days of NPM1 KD (right) conditions. **B** Pie chart depicting the distribution of genomic regions with differential H3K79me2 enrichment upon 3 days of NPM1 KD compared to control (CTR) condition (NPM1 KD/CTR). **C** Heatmap of regions with increased (left) and decreased (right) H3K79me2 enrichment clustered according to the control, NPM1KD, and NPM1KD/control conditions. Data are normalized by sequencing depth and input control as log2(ChIP/Input). The difference between NPM1KD and control conditions was calculated from RPKM normalized bigwig files as log2(NPM1KD/Control). The metaprofiles (top) show the average log2FC (LFC) of each cluster. **D** Dotplot of GO-enrichment analysis of differentially H3K79me2-enriched regions. Gene counts and adjusted p-values are indicated at the right corner and the x-axis depicts gene ratios. Threshold for enrichment analysis was adjusted to p < 0.01 and gene ontology of molecular functions were considered for annotation. **E** Individual data points plot showing the fold change enrichment over control of H3K79me2 calculated after ChIP-qPCR upon 3 days of NPM1 KD (KD/CTR). C3T2.1 is used as negative control region. Statistical analysis was performed on n = 5 biological replicates using an unpaired two tailed t-test. *p < 0.05, **p < 0.01. Error bars represent S.D

We next investigated whether NPM1 KD altered the local distribution of H3K79me2 alongside the global increase of the histone mark. Chromatin immunoprecipitation followed by sequencing (ChIP-seq) with an H3K79me2 specific antibody identified the genomic regions that enriched differentially in H3K79me2 upon NPM1 KD. Two biological replicates were used, and immunoblots verified decreased NPM1 and increased H3K79me2 levels, respectively (Additional file 1: Fig. S3A). In control N2a cells, 44,277 peaks were identified and H3K79me2 was mainly distributed at promoter (~ 38%) and intronic regions (~ 43%), and to a lesser extent at exons (~ 8%) and distal intergenic regions (~ 6%) (Fig. 3A). Upon NPM1 KD, 42,022 peaks were identified and global distribution of H3K79me2 was similar to that of the control dataset (Fig. 3A). Upon analyzing specifically the regions that showed differential enrichment of H3K79me2, we observed that the majority of these regions annotated to promoter regions (~ 85%) (Fig. 3B). We detected 1131 regions with significant changes upon NPM1 KD (FDR < 0.05), 308 of which had increased, and 823 decreased H3K79me2 levels (Fig. 3C). Yet, the observed changes were overall subtle in a range between 1.02 and −0.68 Log2 Fold Change (LFC) (Fig. 3C). We annotated the differentially methylated regions to overlapping or proximal genes (Additional file 2: Table S1). Notably, most genes (11469) retained H3K79me2 upon NPM1 KD and only a minority (484 regions in control and 841 regions in NPM1 KD) showed unique changes in H3K79me2 (Additional file 1: Fig. S3B). Gene Ontology (GO)-term analysis for molecular functions identified, among others, a class of genes involved in chromatin binding, including the epigenetic modifiers *Ezh1* and *Kdm3a* (Fig. 3D and Additional file 1: Fig. S3C). These chromatin binding target genes showing differences in the H3K79me2 level upon NPM1 KD (Additional file 1: Fig. S3D-E), were particularly interesting because of their known impact on nucleoli organization. EZH1 is part of the Polycomb repressive complex 2 (PRC2), which contains in its canonical form the EZH2 histone methyltransferase specific for H3K27me3 [73]. KDM3A is a histone demethylase targeting specifically H3K9me2 [72]. Both H3K27me3 and H3K9me2 enrich at silent heterochromatin surrounding the nucleolus (organized in so-called nucleoli associated domains, NADs) and contribute in this location to preserve nucleoli structure and genome stability [66] (Bersaglieri et al*.*, [7]) (Holmberg Olausson, Nistér and Lindström, [28]). Of further note, we identified the DNA binding factor *Ctcf* among the genes with increased H3K79me2 enrichment upon NPM1 KD (Additional file 1: Fig. S4A). CTCF interacts with NPM1 and participates in the targeting of heterochromatin to the nucleolar periphery [26]. Moreover, a reduction in CTCF expression results in nucleolar fragmentation and reduced rDNA silencing [23].

We used ChIP-qPCR to validate altered H3K79me2 levels at selected chromatin modifiers genes and correlated the impact of H3K79me2 enrichment on gene expression by assessing transcriptional adaptation using RT-qPCR upon NPM1 KD in N2a cells. ChIP-qPCR analysis only returned significantly increased levels for H3K79me2 at the promoter of *Ezh2 and Ctcf*, alongside *Dot1l* and an almost significant enrichment at *Npm1* (Fig. 3E). We noticed, however, strong batch effects in ChIP-qPCRs in our N2a cells that might hide statistically significant effects of NPM1 KD.

**Fig. 4:**
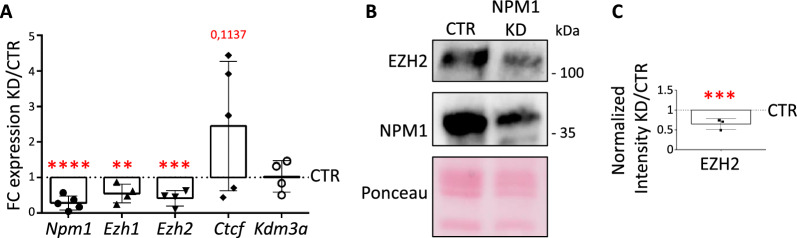
H3K79me2 upregulation correlates with changes in the expression of chromatin binding genes upon NPM1 KD. **A** Individual data points plot showing the fold change (NPM1 KD/CTR) expression of the indicated genes calculated after RT-qPCR upon 3 days of NPM1 KD in N2a cells. *Gapdh* was used as reference gene for normalization. Statistical analysis was performed on n = 5 biological replicates using an unpaired two tailed and one tailed (*Npm1*) t-test; *p < 0.05, **p < 0.01. Outliers were identified using a Grubbs method. Error bars represent S.D. **B** Representative immunoblot analysis of the levels of EZH2 and NPM1 upon NPM1 depletion (NPM1 KD, 3 days) in N2a cells. Ponceau staining was used for total protein normalization. **C** Quantification of the signal intensity of EZH2 shown in B was done using Fiji (ImageJ; bottom). EZH2 levels were first normalized to the corresponding Ponceau signal and then presented as a ratio over the control values. Statistical analysis was performed on n = 3 biological samples using an unpaired two tailed t-test. *p < 0.05, **p < 0.01. Quantification of NPM1 KD shown in Additional file 1: Fig. S4E. Error bars represent S.D

To survey if changes in H3K79me2 levels correlated with altered transcription of chromatin binding genes in N2a cells upon NPM1 KD, we quantified their expression levels by RT-qPCR analysis. Again, the expression of chromatin binding genes was highly variable between batches in this assay, especially for *Ezh1* and *Ezh2*, but *Ctcf and Kdm3a* nevertheless increased significantly upon NPM1 KD (Additional file 1: Fig. S4B)*.* We assumed that the high variability among batches might correlate to variable *Dot1l* expression following NPM1 KD. Indeed, we identified *Ezh1* and *Ezh2* expression correlating with the respective *Dot1l* dose (Additional file 1: Fig. S4C): up to a ^~^2.5 fold increase of *Dot1l* expression upon NPM1 KD, *Ezh1* and *Ezh2* RNA levels decreased significantly (Fig. 4A). Immunoblots indicated significantly reduced EZH2 protein levels upon NPM1 KD (Fig. 4B, C), alongside increased H3K79me2 (Additional file 1: Fig. S4D, E). In contrast, *Dot1l* expression levels higher than 2.5 fold upon NPM1 KD, showed a tendency to increase *Ezh1* and *Ezh2* transcription, although this increase in expression was not statistically significant considering the lower number of samples. Due to this highly variable response to NPM1 KD we cannot definitely conclude about the effect on *Ezh2* expression.

NPM1 plays a key role in ribosome biogenesis, therefore we also analyzed if NPM1 KD would affect rDNA gene expression. We found a significant decrease in the levels of the 28S ribosomal RNA upon NPM1 KD, whereas DOT1L OE did not affect the expression of ribosomal genes (Additional file 1: Fig. S4F). We conclude that modulation of rDNA transcription is a specific function of NPM1 and it is not dependent of DOT1L. This is also in agreement with the preferential localization of DOT1L/NPM1 complex in the nucleus (Fig. 1B, C).

Overall, our data showed that upon NPM1 KD increased levels of H3K79me2 correlated with expression changes of chromatin binding genes, which are involved in the organization of heterochromatin around nucleoli and which contribute to the establishment and/or maintenance of nucleoli architecture.

### NPM1 KD triggers nucleoli fragmentation and represses DNA repeats at peri-nucleolar heterochromatin through H3K27me3

**Fig. 5:**
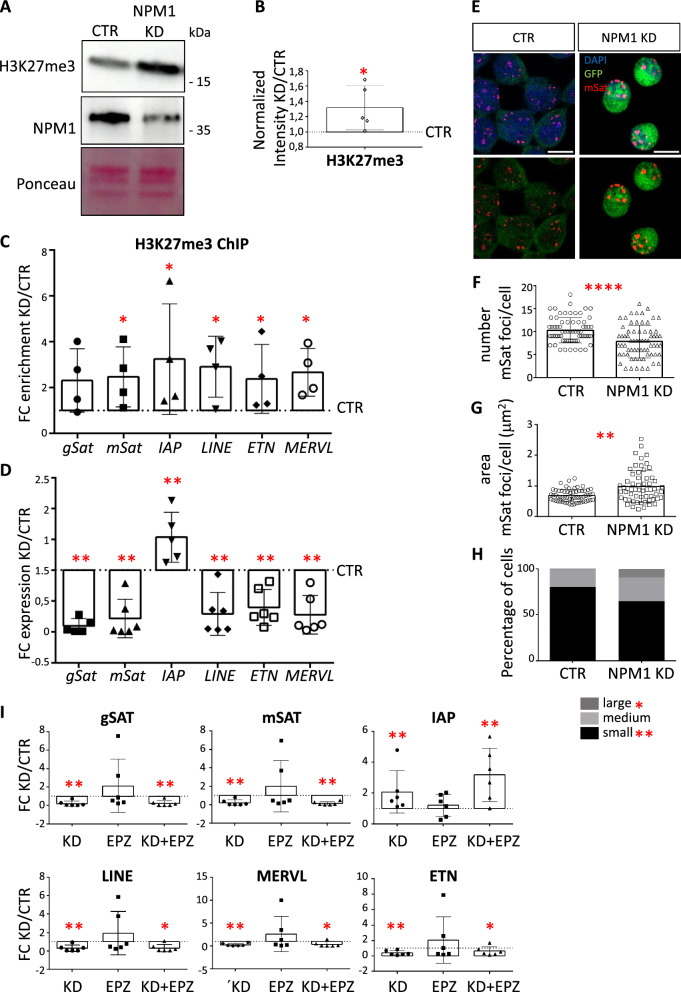
NPM1 KD triggers the silencing of DNA repeats and disrupts the spatial organization of peri-nucleolar heterochromatin. **A** Representative immunoblot analysis of the levels of the H3K27me3 and NPM1 upon 3 days of NPM1 KD in N2a cells. Ponceau staining was used for total protein normalization. **B** Quantification of the signal intensity of H3K27me3 shown in A was done using Fiji (ImageJ). H3K27me3 levels were first normalized to the corresponding Ponceau signal and then presented as a ratio over the control values. Statistical analysis was performed on n = 5 biological samples using an unpaired two tailed t-test. *p < 0.05, **p < 0.01. Error bars represent S.D. **C** Individual data points plot showing the fold change enrichment of H3K27me3 calculated after ChIP-qPCR upon NPM1 KD (KD/CTR, 3 days). *gSAT* (gamma satellites), *mSAT* (major satellites). Statistical analysis was performed on n = 4 biological replicates using an unpaired two tailed t-test. *p < 0.05, **p < 0.01. Error bars represent S.D. **D** Individual data points plot showing the fold change (NPM1 KD/CTR) in the expression of the indicated DNA repeats calculated by RT-qPCR upon 3 days of NPM1 KD in N2a cells. *Gapdh* was used as reference gene for normalization. Abbreviations as in C. Statistical analysis was performed on n = 6 biological replicates using an unpaired two tailed t-test; *p < 0.05, **p < 0.01. Outliers were identified using a Grubbs method. Error bars represent S.D. **E** Confocal immunofluorescence images of immune-FISH performed on N2a cells after 3 days of NPM1 KD using cy3-labeled probes specific for mSat (red). GFP antibody (green) was used to highlight NPM1 KD cells. Scale bars 10μm. **F** Quantification of the average number of mSat foci in control (CTR) and after 3 days of NPM1 KD. Statistical analysis was performed on n = 3 biological replicates and on n = 68 (CTR) and n = 68 (KD) cells using a two-way ANOVA with Sidak’s post-hoc test. *p < 0.05, **p < 0.01. Error bars represent S.D. **G** Quantification of the mean area of mSat foci per cell in control (CTR) and after 3 days of NPM1 KD. Statistical analysis was performed on n = 3 biological replicates and n = 20 nuclei per condition using a two-way ANOVA with Sidak’s post-hoc test. *p < 0.05, **p < 0.01.Error bars represent S.D. **H** Nuclei were divided in 3 categories according to the average size of mSat foci per nuclei: small (0.2–1 μm^2^), medium (1–2 μm^2^) and large (> 2 μm^2^). Statistical analysis was done between classes using a two-way ANOVA with Sidak’s post-hoc test. *p < 0.05, **p < 0.01. Only small and large mSat foci show a significant difference in their average size between control (CTR) and NPM1 KD. **I** Individual data points plot showing the expression of the indicated DNA repeats upon NPM1 depletion (KD), EPZ treatment (EPZ) or EPZ treatment of NPM1 KD (KD + EPZ) in N2a cells. *Gapdh* was used as reference gene for normalization. Statistical analysis was performed on n = 6 biological replicates using an unpaired two tailed Student's t-test; *p < 0.05, **p < 0.01. Error bars represent S.D

As NPM1 KD increased H3K79me2 levels and altered the expression of chromatin modifiers, including EZH2 and KDM3A, we next analyzed H3K27me3 and H3K9me2 global levels by immunoblot in this condition. Upon NPM1 KD, H3K27me3 levels raised (Fig. 5A, B), H3K9me2 levels remained unchanged, while H3K9ac decreased (Additional file 1: Fig. S5A, B). In view of these data together with the enrichment of H3K27me3 at peri-nucleolar heterochromatin [66] (Bersaglieri et al., [7]) (Holmberg Olausson, Nistér and Lindström, [28]), we hypothesized that NPM1 KD might impinge on peri-nucleolar heterochromatin. Thereby, higher levels of H3K27me3 might impair the expression of DNA repeats within this genomic location, and eventually affect heterochromatic organization and nucleoli structure. Indeed, we observed increased H3K27me3 at most DNA repeats upon NPM1 KD using ChIP-qPCR (Fig. 5C), alongside decreased transcription as shown by RT-qPCR (Fig. 5D). Interestingly we also identified increased enrichment of H3K79me2 at DNA repeats (Additional file 1: Fig. S5C), in agreement with the increase of *Dot1l* expression upon NPM1 KD (Fig. 2D). We also examined potential changes in the cellular localization of peri-nucleolar heterochromatin by fluorescence in situ DNA hybridization (DNA FISH) using probes specific for major satellite repeats (mSat) (Fig. 5E). In control (CTR) cells peri-centromeric heterochromatin was organized in distinct foci. Upon NPM1 KD this well-defined organization was resolved. We observed an overall decreased number of foci (Fig. 5F), and the appearance of bigger foci per nucleus (Fig. 5G). Smaller foci were the most affected because upon NPM1 KD the percentage of cells having smaller foci decreased significantly in favor of bigger foci (Fig. 5H). These findings suggested that probably smaller mSat foci tended to converge and fuse. We investigated the possibility that observed alteration of peri-nucleolar heterochromatin organization also affected nucleoli structure upon NPM1 KD. Confocal immunofluorescence using an antibody against the nucleolar marker FBL showed an increased number of nucleoli after NPM1 KD (Additional file 1: Fig. S6A, B).

Our findings of an increased enrichment of H3K79me2 together with increased H3K27me3 levels at peri-nucleolar heterochromatin, let us reason that inhibition of DOT1L enzymatic activity could rescue, at least partially, the effects of NPM1 KD on DNA repeats silencing and nucleoli structural organization. However, treatment of N2a cells with the DOT1L inhibitor Pinometostat (EPZ-5676, (EPZ)) alone had strong and variable batch effects on DNA repeats transcription (Fig. 5I). In combination with NPM1 KD, EPZ treatment was not sufficient to rescue significantly DNA repeats repression (Fig. 5I). DOT1L inhibition alongside NPM1 KD, however, ameliorated the nucleoli fragmentation phenotype, that we observed in the presence of limiting amounts of NPM1 (Additional file 1: Fig. S6A–C).

Together, our results suggested that NPM1 KD triggers global changes in the epigenome, including an increased enrichment of H3K27me3 at peri-nucleolar heterochromatin associated with increased repression of DNA repeats. DNA repeats silencing might contribute to the loss of peri-nucleolar heterochromatin organization and to the nucleoli fragmentation phenotype observed in the presence of limiting amounts of NPM1. However, DOT1L's enzymatic activity seems dispensable for this function.

### NPM1 and DOT1L engage in a regulatory feedback loop

**Fig. 6:**
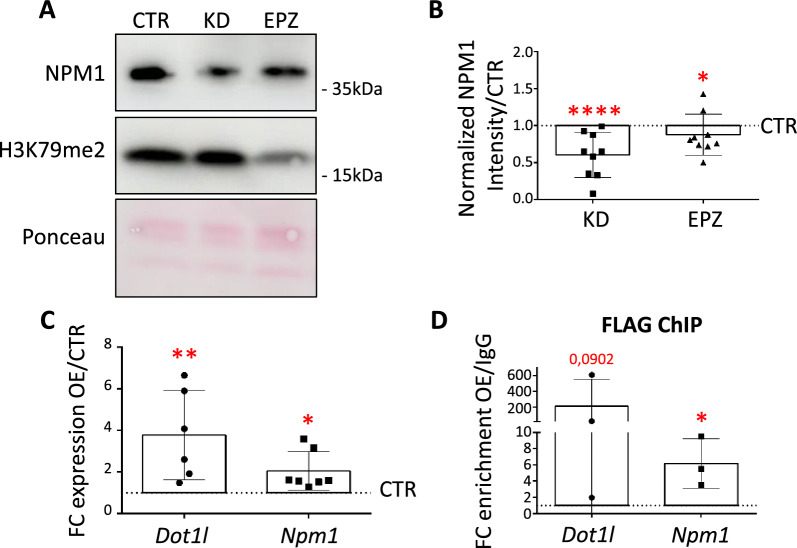
DOT1L and NPM1 engage in a feedback loop regulation. **A** Representative immunoblot analysis of NPM1 expression upon 3 days of NPM1 KD or EPZ treatment (EPZ) in N2a cells. Ponceau staining was used for total protein normalization. **B** Quantification of the signal intensities shown in A. NPM1 levels were first normalized to the corresponding ACTIN signal and then presented as a fold change over the control values in both conditions (Intensity/CTR). Statistical analysis was performed on n = 9 biological replicates using an unpaired two tailed t-test. *p < 0.05, **p < 0.01. Error bars represent S.D. **C** Individual data points plot showing the fold change expression (FC OE/CTR) of DOT1L and NPM1 calculated after RT-qPCR upon 3 days of DOT1L OE in N2a cells. *Gapdh* was used as reference gene for normalization. Statistical analysis was performed on n = 7 biological replicates using a unpaired one tailed (*Dot1*) and two tailed (*Npm1*) t-test. *p < 0.05, **p < 0.01. Outliers were identified using a Grubbs method. Error bars represent S.D. **D** Individual data points plot showing the fold change enrichment of DOT1L (FLAG) over control after by ChIP-qPCR analysis upon 3 days of DOT1L OE in N2a cells. Statistical analysis was performed on n = 3 biological replicates using a paired one tailed t-test: *p < 0.05, **p < 0.01. Error bars represent S.D

Among the genes that enriched for H3K79me2 upon NPM1 KD, we found *Npm1* as well as *Dot1l* itself (Additional file 1: Figs. S4B, S7A-B). This suggested that DOT1L might regulate its own as well as the expression of *Npm1,* and in turn, NPM1 seems to modulate DOT1L expression (Fig. 2D) and/or enzymatic activities as shown by the increased global levels of H3K79me2 upon NPM1 KD (Fig. 2A, B). To get further insights into a possible feedback loop regulation between NPM1 and DOT1L, we treated N2a cells with the DOT1L inhibitor EPZ, which decreased NPM1 protein expression as shown by immunoblot (Fig. 6A, B). Furthermore, testing the effect of overexpression (OE) of DOT1L we observed a significant increase of *Npm1* transcription (Fig. 6C). The effect of DOT1L OE on NPM1 protein levels was less clear because of strong batch effects (Additional file 1: Fig. S7C-D). ChIP-qPCR confirmed the presence of DOT1L-FLAG in N2a cells at both *Npm1* as well as *Dot1l* promoters upon OE (Fig. 6D). Together these results suggested that DOT1L is recruited to the *Npm1* promoter and that DOT1L enzymatic activity is involved in regulating *Npm1* expression.

We further investigated if variable levels of DOT1L would also affect NPM1 localization and/or appearance of nucleoli. By employing immunofluorescence staining of N2a cells, we did neither observe changes in NPM1 localization upon DOT1L OE (Additional file 1: Fig. S7E), nor altered NPM1 subcellular localization upon DOT1L inhibition with EPZ (Additional file 1: Fig S7F). We concluded that, at least for the variables studied, neither DOT1L OE nor its enzymatic activity alters NPM1 localization globally, but that DOT1L and NPM1 engage in a feedback loop regulation where NPM1 curbs DOT1L enzymatic activity by lowering its expression level and DOT1L modulates NPM1 expression levels in an H3K79me-dependent manner.

### DOT1L controls DNA repeats expression to maintain peri-nucleolar heterochromatin organization

**Fig. 7:**
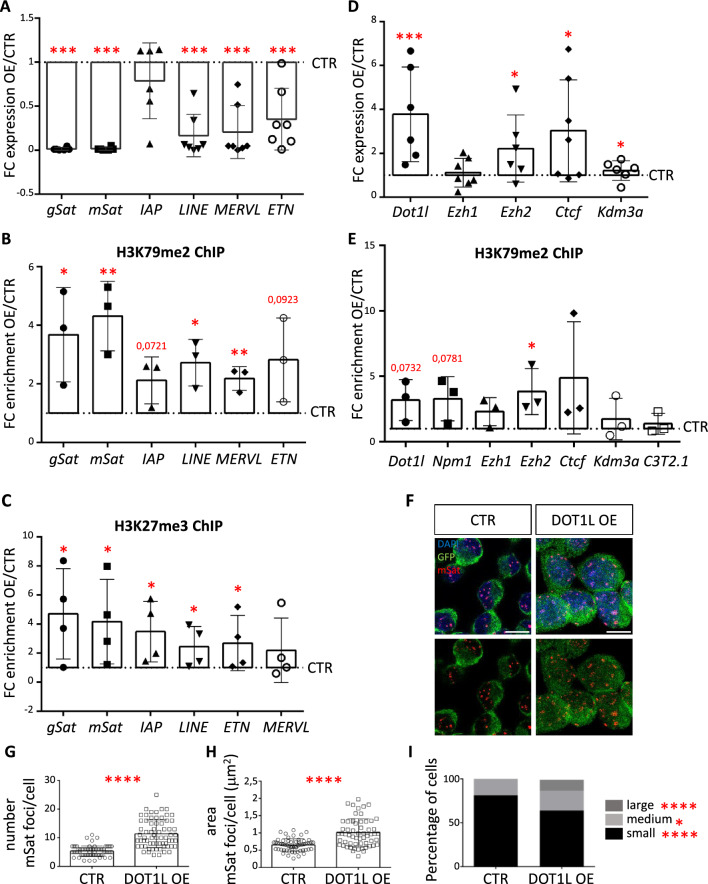
Increased DOT1L levels are responsible for DNA repeat repression at peri-nucleolar heterochromatin. **A** Individual data points plot showing the fold change expression (FC OE/CTR) of the indicated DNA repeats after RT-qPCR upon 3 days of DOT1L OE in N2a cells. *Gapdh* was used as reference gene for normalization. Abbreviations are: gSAT (gamma satellites), mSAT (major satellites). Statistical analysis was performed on n = 7 biological replicates using an unpaired two tailed t-test. *p < 0.05, **p < 0.01. Outliers were identified using a Grubbs method. Error bars represent S.D. **B** Individual data points plot showing the fold change enrichment over control of H3K79me2 after ChIP-qPCR upon 3 days of DOT1L OE (OE/CTR). Abbreviations as in A. Statistical analysis was performed on n = 3 biological replicates using an unpaired two tailed Student's t-test. *p < 0.05, **p < 0.01. Error bars represent S.D. **C** Individual data points plot showing the fold change enrichment over control of H3K27me3 at the indicated DNA repeats after ChIP-qPCR upon 3 days of DOT1L OE (OE/CTR). Abbreviations as in A. Statistical analysis was performed on n = 4 biological replicates using an unpaired two tailed t-test. *p < 0.05, **p < 0.01. Error bars represent S.D. **D** Individual data points plot showing the fold change expression (FC OE/CTR) of the indicated genes after RT-qPCR upon 3 days of DOT1L OE in N2a cells. *Gapdh* was used as reference gene for normalization. Statistical analysis was performed on n = 7 biological replicates using an unpaired one tailed (*Dot1l*) and two tailed t-test (all other genes). *p < 0.05, **p < 0.01. Outliers were identified using a Grubbs method. Error bars represent S.D. **E** Individual data points plot showing the fold change enrichment over control of H3K79me2 at the indicated genes, calculated by ChIP-qPCR upon 3 days of DOT1L OE (OE/CTR). C3T2.1 is used as negative control gene. Statistical analysis was performed on n = 3 biological replicates using an unpaired two tailed Student's t-test. *p < 0.05, **p < 0.01. Error bars represent S.D. **F** Confocal immunofluorescence images of immune-FISH performed on N2a cells after 3 days of DOT1L OE using cy3-labeled probes specific for mSat (red). GFP antibody (green) was used to highlight cells with DOT1L OE. Scale bars 10μm. **G** Quantification of the average number of mSat foci in control (CTR) and after 3 days of DOT1L OE. Statistical analysis was performed on n = 3 biological replicates and on n = 68 (CTR) and n = 68 (OE) cells using a two-way ANOVA with Sidak’s post-hoc test. *p < 0.05, **p < 0.01. Error bars represent S.D. **H** Quantification of the mean area of mSat foci per cell in control (CTR) and after 3 days of DOT1L OE. Statistical analysis was performed on n = 3 biological replicates and n = 20 nuclei per condition using a two-way ANOVA with Sidak’s post-hoc test. *p < 0.05, **p < 0.01. Error bars represent S.D. **I** Nuclei were divided in 3 categories according to the average size of mSat foci per nuclei: small (0.2–1 μm^2^), medium (1–2 μm^2^) and large (> 2 μm^2^). Statistical analysis was done between classes using a two-way ANOVA with Sidak’s post-hoc test. *p < 0.05, **p < 0.01

Peri-nucleolar heterochromatin correlates with low gene density and transcriptional repression and undergoes bursting of transcriptional activation, which is necessary for its repressive function. Transcriptional bursting requires the presence of DOT1L [42]. As *Dot1l* expression (Fig. 2D) and H3K79me2 levels increased upon NPM1 KD (Fig. 2A-C), we investigated the possibility that increased levels of DOT1L would be responsible for the aberrant repression of DNA repeats (Fig. 5D) and the loss of peri-nucleolar heterochromatin organization (Fig. 5E–H), which we observed upon NPM1 KD. To mimic the DOT1L increase observed upon NPM1 KD (Fig. 2D) without affecting NPM1 protein levels at the same time, we used DOT1L OE in N2a cells (Additional file 1: Fig. S8) and measured DNA repeats expression by RT-qPCR. Similar to NPM1 KD (Fig. 5D), we observed a drastic reduction in the expression of DNA repeats (Fig. 7A) upon DOT1L OE. H3K79me2 and H3K27me3 levels increased at DNA repeats chromatin upon DOT1L OE as measured by ChIP-qPCR (Fig. 7B, C), also in accordance with the effects observed upon NPM1 KD (Fig. 5C). NPM1 KD displayed a strong batch effect towards *Ezh2* expression (Additional file 1: Fig. S4C), which seemed to relate to the level of DOT1L, whereby a strong increase in DOT1L expression correlated with increased levels of *Ezh2* transcription (Additional file 1: Fig. S4D). Indeed, OE of DOT1L increased *Ezh2* expression (Fig. 7D). ChIP-qPCR upon DOT1L OE revealed increased H3K79me2 levels at the *Ezh2* promoter (Fig. 7E). Moreover, similarly to NPM1 KD (Fig. 5E), DOT1L OE altered the appearance of mSat as measured by DNA FISH (Fig. 7F). In contrast to NPM1 KD, DOT1L OE increased the number of mSat foci, which might indicate that in total more mSat sequences were accessible to the probes (Fig. 7G). In line with NPM1 KD, DOT1L OE also caused clustering/fusion of mSat as indicated by an increased size of foci, and an increased number of larger foci, on the expenses of smaller ones (Fig. 7H, I). Our findings indicated that increased levels of DOT1L, similarly to NPM1 KD, silenced DNA repeats and affected the organization of peri-nucleolar heterochromatin, a process that involved activating EZH2 expression and increasing H3K27me3 levels.

## Conclusions

**Fig. 8:**
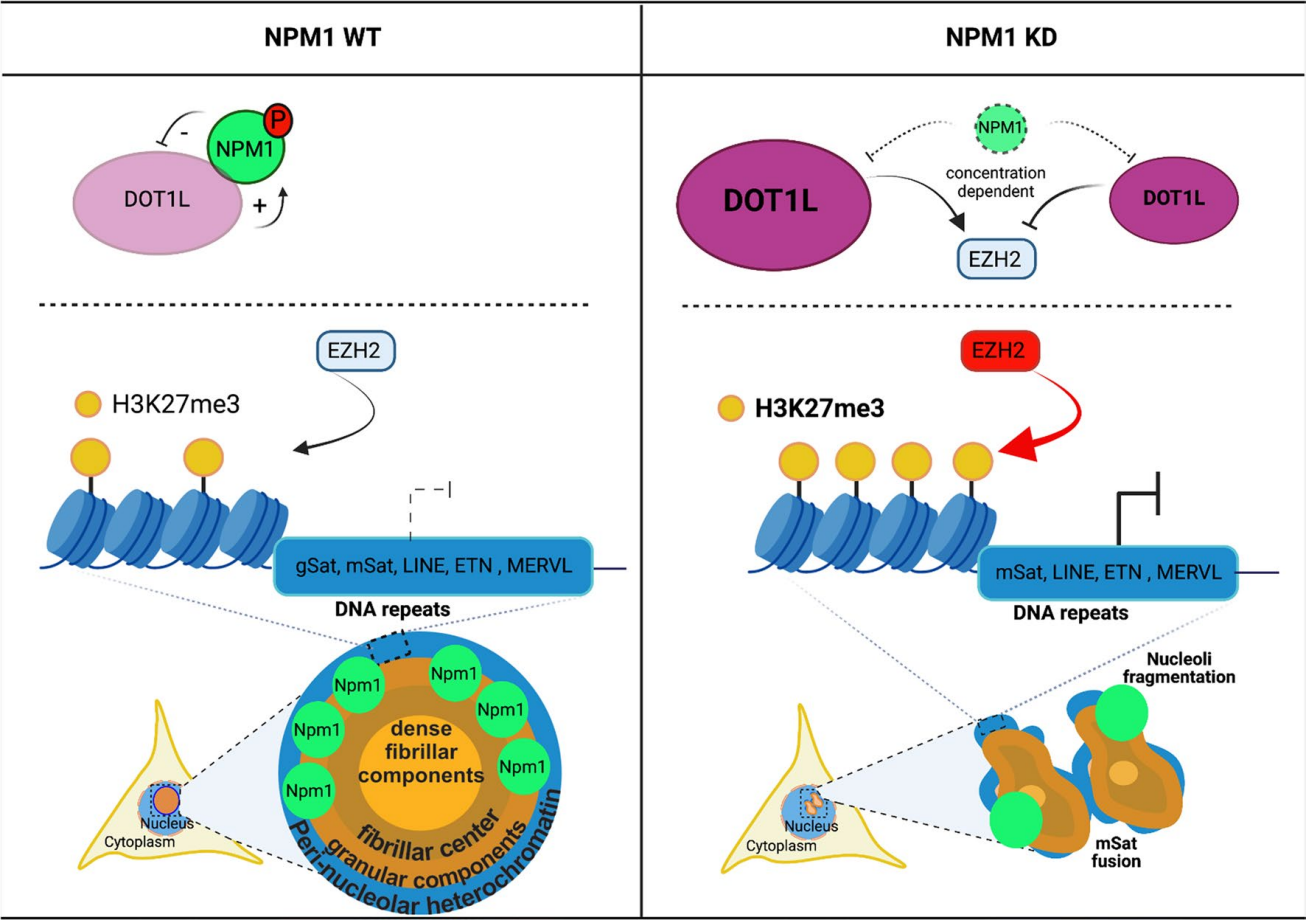
Cartoon model of NPM1 and DOT1L functions in regulating their expression and in heterochromatin organization around the nucleoli. **Left:** WT situation in which NPM1 restricts DOT1L expression levels and DOT1L promotes NPM1 expression. Moderate expression of DNA repeats in peri-nucleolar heterochromatin is accompanied with moderate levels of H3K27me3, mediated by the PRC2 member EZH2. Nucleoli integrity and peri-nucleolar heterochromatin is preserved by expression of NPM1. **Right:** NPM1 KD leads to increased levels of DOT1L expression and H3K79me2. Both, NPM1 and DOT1L regulate *Ezh2* transcription, whereby its levels depend on DOT1L concentrations. NPM1 KD alike DOT1L OE increase H3K79me2 and H3K27me3 levels at DNA repeats and repress basic expression of the DNA repeats. In addition to silencing of the DNA repeats in the peri-nucleolar heterochromatin through an altered epigenome, the structure of the heterochromatin and of the nucleoli is also affected towards larger DNA repeat foci and fragmented nucleoli

Our data showed that in N2a cells monomeric NPM1 interacts with DOT1L at the chromatin level outside the nucleoli. Being short in attributing specific functions for the NPM1/DOT1L complex itself, we hypothesize that the interaction between NPM1 and DOT1L might be required to maintain homeostatic levels of both proteins in a feedback loop regulation. On the level of action as individual, though functionally synergizing proteins, we propose a role in the perseverance of peri-nucleolar heterochromatin organization around the nucleoli: NPM1 constrains DOT1L enzymatic activity, whereas DOT1L is recruited to the *Npm1* gene promoter to activate its expression. Increased levels of DOT1L upon NPM1 KD correlate with increased H3K79me2 levels and associated transcriptional changes in the expression of chromatin binding genes including *Ezh2*. Moreover, NPM1 knockdown caused H3K27me3 enrichment at DNA repeats within peri-nucleolar heterochromatin and enhanced repeat silencing. The impact of DOT1L on DNA repeats transcription is seemingly independent of its enzymatic activity and might be propagated through a DOT1L scaffolding function. The observed changes in DNA repeats expression, however, correlate well with the loss of heterochromatin organization around the nucleoli and might contribute to the nucleoli fragmentation occurring upon NPM1 reduction (Fig. 8).

## Discussion

### Diverse regulative layers of NPM1 and DOT1L interaction

Our data show that NPM1 interacts with DOT1L in the neuroblastoma N2a cell line. With our finding we provide an extension of the previously shown protein association between NPM1 and DOT1L in HEK293T cells [49], suggesting that this interaction might be of broader and functional relevance because it occurs in diverse cellular contexts. The conserved nature of NPM1/DOT1L interaction suggests that both proteins are implicated in crucial aspects of the molecular and epigenetic mechanisms of nucleoli organization, most likely in different cell types, as we have unraveled here. We highlight further that DOT1L interacts exclusively with the monomeric form of NPM1, which is mainly present in the nucleus. In addition, the NPM1/DOT1L complex is excluded from the nucleoli, in which NPM1 is more abundant compared to other cellular compartments. Of note, monomeric NPM1 is highly phosphorylated at specific sites and some forms of phospho-NPM1 localize preferentially in the nucleus [34, 44]. Interestingly, a similar dependence on the phosphorylation state has been observed for DOT1L. DOT1L is phosphorylated by CDK1 (cyclin dependent kinase 1) and 2 [14]. Phosphorylation of DOT1L by CDK1 and CDK2 in mESC inactivates its H3K79me2 enzymatic activity and leads to translocation of DOT1L into the cytosol [43]. Thus, although we did not address this issue, it is tempting to speculate that phosphorylation is a means to control the interaction of NPM1 and DOT1L, and that this is one of the mechanisms through which NPM1 and DOT1L functions can be controlled.

Moreover, in cancer cells, an acetylated form of NPM1 (acNPM1) functions as a coactivator of RNA polymerase II-dependent transcription to promote the expression of genes involved in tumorigenesis [59]. Given that both, NPM1 and DOT1L are modified through acetylation [41], it is tempting to speculate that in addition to phosphorylation, other posttranslational modifications like acetylation might influence NPM1/DOT1L complex formation, and/or regulate their functions separately from each other.

NPM1/DOT1L binding is also influenced by RNA, because RNase treatment increased the association of the two partners. RNA as critical regulator of NPM1/DOT1L interaction is further supported by our finding of a preferential interaction of DOT1L with monomeric NPM1. RNA prevents NPM1 oligomerization and translocation into the nucleoli, which might result in increased amounts of NPM1 in the monomeric form that is consequently available for interaction with DOT1L (Okuwaki, Tsujimoto and Nagata, 2002).

### NPM1 and DOT1L are engaged in feedback loop regulation

A further crucial finding of our study is the existence of a regulatory feedback loop that involves both NPM1 and DOT1L. In this context, NPM1 represses *Dot1l* transcription, whereas DOT1L is recruited to the *Npm1* promoter and its enzymatic activity is required to activate NPM1 expression. Our study did not unravel as of yet whether NPM1 and DOT1L proteins act independently or as a complex to target their own genes. However, this assumption is not unlikely, as a direct role of NPM1 as a transcriptional regulator has been recently shown in NPM1c +AML [68].

The significance of this regulatory mechanism in which NPM1 and DOT1L influence the expression of their own genes is underlined by the importance of NPM1 and DOT1L homeostasis for cell viability and development. NPM1 overexpression is considered a prognostic marker for the recurrence and progression of various hematological malignancies [13] and high levels of NPM1 have been observed in specific areas of the brain in a mouse model of Huntington´s disease [50]. Moreover, DOT1L depletion as well as DOT1L mutations that alter its activity and abundance have been implicated in neurodevelopmental diseases including microcephaly, and different types of cancer [21, 63, 78]. Given these data, the maintenance of physiological levels of NPM1 and DOT1L seems to be crucial for cell survival and it might be achieved in some contexts by engaging both NPM1 and DOT1L in a common regulatory feedback loop.

### NPM1 depletion alters the epigenome through changes in the expression of chromatin binding genes

In the presence of limiting amounts of NPM1, H3K79me2 enrichment at the promoter of chromatin binding genes correlates with variable changes in their expression. Although we did not detect strong changes in the transcription of DOT1L target genes upon NPM1 KD, these subtle changes are expected in agreement with DOT1L being a modulator of transcription rather than a transcriptional activator [2, 20, 21]. Seemingly, H3K79me2 increased at the promoter of *Ezh2* upon NPM1 KD (Fig. 3E). However, we observed highly variable *Ezh2* expression changes in different experiments (Additional file 1: Fig S4B). In contrast to this finding, DOT1L OE increased expression of the PRC2 component (Fig. 7D). Through pairwise comparisons of data points upon NPM1 KD, we correlated the *Dot1l* dose and *Ezh1* and *Ezh2* levels. We noticed that high levels of *Dot1l* correlated with increased levels, whereas lower levels of *Dot1l* with decreased levels of the PRC2 components (Additional file 1: Fig. S4C). In agreement with the potential role of DOT1L in the transcriptional regulation of *Ezh2* it was previously shown that DOT1L and EZH2 functionally converge to prevent the premature differentiation of activated B cells into plasma cell [3]. As the NPM1 KD produced variability in *Dot1l* expression levels, the dose dependency in *Ezh1* and *Ezh2* regulation can, at least partially, account for the substantial variability in the expression of these chromatin binding genes in response to NPM1 KD. In addition, (i) the different efficiency of NPM1 KD in each biological replicate, (ii) batch effects across experimental conditions, and (iii) the heterogeneous nature of the N2a cells might contribute as well. Given the high variability in these experiments, we cannot conclude that the increased global levels of H3K27me3 depend alone on increased EZH2 levels, although its expression is altered upon NPM1 KD. It is still possible that the increase of H3K27me3 at peri-nucleolar DNA repeats occurs independently of changes in EZH2 protein levels. One alternative scenario could be that upon NPM1 KD, DOT1L recruitment at peri-nucleolar heterochromatin affects chromatin accessibility, favoring binding of EZH2, and increasing H3K27me3 at DNA repeats, independent from a direct transcriptional control of *Ezh2*. In this regard, we have previously demonstrated that in neural progenitor cells, DOT1L inactivation leads to reduced H3K27me3 levels at selected target genes, without impacting *Ezh2* expression, but by altering PRC2 recruitment [2]. Alternatively, it is also possible that EZH1 replaces EZH2 functions and increases H3K27me3 at non canonical target genes [37].

Our ChIP-seq analysis unraveled more regions with decreased than increased levels of H3K79me2 (Fig. 3C), yet overall H3K79me2 is increased after NPM1 KD (Fig. 2A). We think that this apparent discrepancy might be due to the different approaches we used to analyze H3K79me2 levels upon NPM1 KD. In the ChIP-seq we specifically focused on the H3K79me2 incorporated into chromatin at a certain time. In the immunoblot analysis, however, we used total cell extract and therefore detected both nuclear and chromatin associated H3K79me2. The nuclear fraction might consist of both H3K79me2 nucleosomes exchanged from regions with decreased H3K79me2 (no specific demethylase for H3K79me2 has been identified so far) as well as from newly modified histones. Those histones are then incorporated into regions with increased H3K79me2 enrichment. Another possible explanation is that the kinetic of H3K79me2 histone loss is faster than their incorporation into chromatin and thus in the ChIP-seq we identify a higher number of regions with decreased levels of H3K79me2. However, we cannot exclude that indirect mechanisms might also take place and that e.g. NPM1 KD could lead to decreased transcription of targets, which would result in decreased H3K79me2 at these genes. We did not address here the role of H3K79me3, which is also a product of DOT1L activity, in peri-nucleolar heterochromatin organization and nucleoli structure. However, also in view of previous findings showing the specific enrichment of this mark at DNA repeats in mESC [42], we cannot exclude its specific contribution to the phenotypes we observed in the absence of NPM1.

### NPM1 and DOT1L impact the expression and organization of DNA repeats at peri-nucleolar heterochromatin

One of the striking features of NPM1 depletion in N2a cells is the loss of heterochromatin organization and nucleoli fragmentation. Specifically, mSat heterochromatin converged into larger foci and their average number per cell decreased. Rearrangements in the organization of the nucleoli structure have been previously observed in fibroblasts and cancer cells as well as in oocytes lacking NPM1 or its isoforms (Holmberg Olausson, Nistér and Lindström, 2014) [9]. These nucleolar phenotypes are at least partly a result of the loss of proper phase separation due to reduced nucleolar NPM1 [29]. However, besides the phenotypic observation, the dynamics between NPM1 expression and heterochromatin organization, and to what extent the functions of NPM1 and DOT1L are involved in the nucleolar phase separation, have not been addressed so far.

In addition, we detected changes in peri-nucleolar heterochromatin activity that is, the enhanced silencing of repetitive regions in NPM1-depleted cells compared to controls. Heterochromatin surrounding the nucleoli consists of repetitive major satellite (pericentric) and minor satellite (centric) DNA sequences and of tandemly repeated ribosomal RNA (rRNA) genes [24]. Correct silencing of these DNA repeats is of key importance to preserve genome stability [40]. Recently, it has been shown that initiation and maintenance of silencing at repetitive regions within peri-nucleolar heterochromatin require a periodical burst of transcription, which is in part dependent on DOT1L [42]. In support of this, the requirement of DNA repeats transcription in heterochromatin formation has been widely confirmed in different species (Barutcu, Blencowe and Rinn,[6]) [64]. Moreover, it has been shown that NPM1 recruitment to peri-nucleolar heterochromatin and proper organization of heterochromatin around nucleoli depends on an architectural RNA component [62]. In agreement with these findings, DOT1L OE in N2a cells increased H3K79me2 and H3K27me3 levels at DNA repeats and impaired their expression as well as mSat organization around nucleoli, similarly to NPM1 KD. However, DOT1L OE did not result in nucleoli fragmentation, as NPM1 KD does. Moreover, inhibition of DOT1L enzymatic activity with EPZ was not sufficient to re-establish correct DNA satellite expression. This suggests to us that NPM1 majorly controls nucleoli integrity and preservation of peri-nucleoli heterochromatin, whereas DOT1L has a modulatory role in this setting, and might be under control of NPM1. DOT1L modulates DNA repeats expression and their spatial organization within the nuclei, and its inhibition is a means to prevent fragmentation of the nucleoli upon NPM1 KD. However, DOT1L effects are mild compared to NPM1, which seems to be the major driving force for preserving nucleoli architecture. However, our data might underestimate the role of DOT1L in this setting, because the presence of NPM1 in our experimental conditions during DOT1L OE could mask a potential direct and impactful role of DOT1L.

Our findings of a non-canonical role of DOT1L in DNA repeats repression are supported by the recent discoveries of Zhao et al. [80], who showed that DOT1L cooperates with NPM1 to silence MERVL expression in mESC. However, repression of MERVL in mESC required DOT1L enzymatic activity. Recently, Malla et al. came to the same conclusion and showed that DOT1L promotes transcription at major satellite repeats in mESC and that DOT1L enzymatic activity is required for this function [42]. In view of these findings, we believe that DNA repeats regulation by DOT1L occurs at multiple levels and it is context-dependent in pluripotent versus more differentiated cells. In this regard, in N2a cells DOT1L scaffolding function rather than its enzymatic activity is key for this function. Thus, differences in DOT1L interactome in N2a cells versus mESC could justify the opposite modulation of DNA repeats expression and the different molecular mechanisms employed.

Our findings of the critical role of the NPM1 and DOT1L in peri-nucleolar heterochromatin functions and organization in N2a cells sheds new light on the versatile roles of NPM1 and DOT1L in chromatin regulation. Moreover, our data advance our understanding of how NPM1 and DOT1L might be recruited to specific target regions, i.e. through interaction with specific protein partners.

Despite much more research is needed to gain full insights into how NPM1 cooperates with DOT1L to impact nucleoli activity, and into the full functional significance of the NPM1/DOT1L protein complex, our findings provide a valuable resource for future studies on how the epigenome contributes to the organization of the nucleolus.

## Material and methods

### N2a cell culture and transfection

Mouse neuroblastoma cell line, Neuro-2a (N2a) was cultured and maintained at 37 °C, 95% relative humidity and 5% CO2 in Dulbecco’s modified Eagle’s medium (DMEM, Gibco) supplemented with 10% fetal bovine serum (FBS, Gibco), 1% non-essential amino acids (NEAA, Gibco), 1% L-glutamine, and 1% penicillin, streptomycin, and neomycin (PSN, Gibco). Cells were seeded in 24 wells plates on coverslips either for proximity ligation assay (PLA) immunofluorescence and DNA-FISH, or in 6 wells plates for biochemistry and ChIP/RT-qPCR. Cells were transfected with Lipofectamine LTX according to the manufacturer´s instructions (ThermoScientific). For NPM1 knockdown (NPM1 KD) and DOT1L overexpression (DOT1L OE) experiments, N2a cells were selected with 9.3 µg/ml Puromycin starting 24 h after transfection and for the remaining time until fixation or collection. Where indicated 10 μM EPZ5676 (Active Biochemicals) in dimethyl sulfoxide (DMSO) (Sigma-Aldrich) was added to N2a cells for 72 h and refreshed every second day. DMSO was used as control treatment. The following plasmids were used for transfection: pLKO.1-shCTR (Sigma, Non Target #3), pLKO.1-shNPM1 (Sigma, TRCN0000115430), pLenti-CMV-HA-2A-GFP (Genscript), pLenti-CMV-DOT1L-FLAG-HA-2A-GFP (Genscript) and pVB-DOT1L-FLAG (Vector Builder).

### Cell lysis, co-immunoprecipitation and immunoblots

For total cell lysate preparation frozen cell pellets were lysed in Lysis Buffer (10 mM Tris pH8, 1 mM EDTA, 1%SDS) supplemented with complete Protease Inhibitor cocktail (Roche-Diagnostics). Cells in Lysis Buffer were kept on ice for 30 min and chromatin disruption was done by sonication for 4 min in AFA 130 μl tubes (Covaris) using the Covaris E220. Cell debris was removed by centrifugation at 14.000 rpm 4 °C 5 min. The supernatant was collected and protein concentrations were quantified photometrically with Bio-Rad Protein Assay Dye Reagent Concentrate (Bio-Rad). 5–15 μg of cell lysates were loaded on 4–20% gradient SDS-PAGE (Bio-Rad). Proteins were transferred to PVDF membranes or to nitrocellulose membranes for Ponceau staining using the Trans-blot Turbo Transfer System (Bio-Rad) following the manufacturer’s instructions. Upon transfer, membranes were further processed for immunoblot following standard procedures.

For co-IP experiments, the cell pellet was resuspended in co-IP Buffer 1 (20 mM Tris pH 7.5, 1 mM EDTA, 100 mM NaCl, 0.5% NP40) and cell lysate was left 30 min on ice. Debris was removed by centrifugation at 14.000 rpm 4 °C 5 min. The cell lysate was incubated with 2 μg of the indicated antibody bound to protein G dynabeads (1 μg antibody/10 μl beads, Invitrogen) overnight. The immunocomplexes were then washed with co-IP Buffer 1 three times and separated by SDS-PAGE. Immunoblotting was performed following standard procedures.

For endogenous NPM1/DOT1L co-IP, cell pellet (4*10^7^ cells) was resuspended in co-IP Buffer 2 (20 mM Hepes pH8, 300 mM NaCl, 0.5% NP40, 10% glycerol, 1 mM EDTA) and the cell lysate was left 30 min on ice. Debris was removed by centrifugation at 14.000 rpm 4 °C 5 min. The cell lysate was equilibrated to 150 mM NaCl by adding drop by drop co-IP Buffer 2 without NaCl. 2 μg of the indicated antibody bound to protein G dynabeads (1 μg antibody/10 μl beads, Invitrogen) was added overnight and cell immunocomplexes were further treated as described before.

For NPM1 cross-linking with EGS (ethylene glycol bis(succinimidyl succinate), ThermoScientific) N2a cells were transfected using Lipofectamine 2000 and allowed to express DOT1L-HA-FLAG for 72 h. The cells were then lysed in co-IP Buffer 1 and incubated with either DMSO or 0.5 mM EGS for 30 min at 25 °C. Crosslinking was quenched with the addition of 0.025 mM Tris–HCl pH 7.5 for 15 min at 25 °C. DOT1L immunoprecipitation was performed using HA antibody as described above.

For nucleic acid digestion, the cell extract in co-IP Buffer 1 was treated with 20 μg of RNase (Promega) or 5U of DNase (Promega) for 30 min before performing the co-IP as described above.

Cell fractionation was performed by resuspending cell pellets in 500 μl Lysis Buffer (15 mM HEPES pH7.5, 10 mM KCl, 5 mM MgCl2, 0.1 mM EDTA, 0.5 mM EGTA, 250 mM Sucrose, 0.4% Igepal, 1 mM DTT, Protease Inhibitor cocktail) followed by incubation on ice for 20 min. Nuclei were centrifuged at 2.000 rpm for 10 min at 4 °C and the supernatant was collected as the cytoplasmic fraction. Nuclei were then resuspended in 100 μl Nuclei Lysis Buffer (10 mM HEPES pH 7.5, 0.1 mM EDTA, 0.1 mM EGTA, 1 mM DTT, Protease Inhibitor cocktail) and incubated on ice for 5 min. Nuclei were pelleted at 15.000 rpm for 5 min at 4 °C and the supernatant was removed as the nucleoplasm fraction. The volume of the nucleoplasm fraction was adjusted to 250 μl with co-IP Buffer (20 mM Tris pH 7.5, 1 mM EDTA, 100 mM NaCl, 0.5% NP40) with Protease Inhibitor cocktail. The chromatin pellet was then resuspended in 250 μl co-IP Buffer (20 mM Tris pH 7.5, 1 mM EDTA, 100 mM NaCl, 0.5% NP40) with Protease Inhibitor cocktail and sonicated with the use of Bioruptor (Diagenode) with the following setting: cycle number: 9 + 9 Time ON: 30 s Time OFF: 45 s. The chromatin and nucleoplasm fraction were the used for co-IP as described before.

The following antibodies were used for co-IP or immunoblots: anti-DOT1L (1:1000, rabbit #77087 Cell Signaling), anti-DOT1L (1:1000, rabbit #90878 Cell Signaling), anti-HA-tag (1:1000, rabbit, #3724, Cell Signaling), anti-H3 (1:1000, goat, ab12079, Abcam), anti-NPM1 (1:1000, mouse, ab10530, Abcam), anti-GAPDH (1:3000, mouse, ab8245, Abcam), anti-Actin ( 1:5000, rabbit, ab179467, Abcam) anti-H3K79me2 (1:1000, rabbit, ab3594, Abcam), anti-H3K27me3 (1:1000, mouse, ab6002, Abcam), anti-H3K9me2 (1:1000, rabbit, ab1220, Abcam), anti-H3K9ac (1:1000, rabbit, ab4441, Abcam). Densitometric analyses were done with ImageJ software.

### Immunofluorescence in cultured cells

Cells were seeded in multi-well plates and transfected as described before. Medium was removed and cells were washed 3 times with PBS. Fixation was performed with 4% paraformaldehyde (PFA, Life Technologies) in PBS for 15 min at RT. After PFA removal, cells were washed 3 times in PBS. Cells were permeabilized with 0.1% Triton-X100/PBS for 15 min and blocked in 10% horse serum/PBS 1 h. Primary antibody incubation was done overnight at 4 °C in blocking solution. Cells were washed 3 times in PBS and incubated with fluorescent secondary antibodies in blocking solution at room temperature for 2 h. After washing 3 times with PBS, cells were incubated for 5 min with DAPI (4′,6-diamidino-2-phenylindole, ThermoScientific) and washed 3 times in PBS. Coverslips were mounted on glass slides with fluorescent mounting medium (#S3023, DAKO). The following first and secondary antibodies were used: LAMINB1 (1:250, rabbit ab133741, Abcam), FIBRILLARIN-Alexa488 (1:250, rabbit, ab184817, Abcam) H3K79me2 (1:250, rabbit, ab3594, Abcam), NPM1 (1:1000, mouse, ab10530, Abcam), GFP (1:250, rabbit, ab290, Abcam), donkey-anti-goat-Alexa488 (1:500, A-11055, Life-Technologies) or -Cy3 (1:500, 705-165-147, Dianova), donkey-anti-rabbit-Alexa488 (1:500, 711-545-152, Dianova) or -Alexa594 (1:500, 711-585-152, Dianova), donkey-anti-chicken-Alexa488 (1:500, 703-545-155, Dianova), donkey-anti-mouse-Alexa488 (1:500, 715-545-151, Dianova) or -Alexa594 (1:500, 715-585-151), donkey-anti-rat-Alexa488 (1:500, 712-545-153, Dianova) or -Alexa594 (1:500, 712-585-153, Dianova) and donkey-anti-rat-AMCA (1:200, 712-155-153, Dianova).

### Immuno-FISH

The immune-FISH cells were initially treated as for the immunofluorescence experiment with the following changes: PFA-fixed cells were permeabilized with 0.2% TritonX100 in PBS, 10 min, then wash with 1 × PBS, 3 times, 5 min; primary and secondary antibody were incubated in PBG 1 × blocking buffer (0,2% Fish Gelatin (#G7041, Sigma) and 0,5% BSA in PBS). After washing out the secondary antibody with 1 × PBG, twice, 5 min and with 1 × PBS, twice, 5 min, cells were fixed again with PFA 4% (Life Technologies) with triton 0.1%, 10 min RT, followed by incubation with glycine 10 mM in H2O, 30 min, RT. Cells were then washed with 1 × PBS, 3 times, 5 min. For each coverslip 20 μl of hybridization mixture (Formamide 70%, Tris HCl pH 7.4 10 mM, blocking reagent 1x (#11096176001, Roche)) containing 0.5 μM of each DNA probe for major satellite repeats (mSat 3a/3b IDT, Additional file 3: Table S2) were added on a glass slide and the coverslips transferred carefully on the drop without making bubbles. The slide was put directly on a metal thermo block at 80 °C, 5 min and then hybridized in a humidified chamber, 2 h, RT. The coverslips were then put back in a 12 wells plate. Washed with Wash solution I (Formamide 70%, Tris HCl 15 mM pH 7.4, BSA 0.15%), twice, 15 min; with Wash solution II (Tris HCl 0.1 M pH 7.4, NaCl 150 mM, Tween 20 0.1%), 3 times, 5 min and incubated with DAPI, 2 min, RT. After a brief wash with 1 × PBS coverslips were mounted with mounting medium (#S3023, DAKO). The following primary and secondary antibody were used: GFP (1:500, cicken, ab13970, Abcam), donkey-anti-chicken-Alexa488 (1:500, 703-545-155, Dianova).

### Image acquisition, quantification and analysis

Images were taken in a Leica Sp8 confocal microscope with a 63X oil objective and a zoom factor of × 3 when indicated. The mean area and number of FBL (FIBRILLARIN) nucleolar stained structures and the mSat foci were quantified using ImageJ with the ‘Analyze particle’ option. Particle size was set at 0.2-3000µm^2^. A two-way ANOVA with Sidak’s post-hoc test was performed with GraphPad Prism Version 6.07 to assess for statistical significance.

### Proximity ligation assay (PLA)

PLA was performed with the Duolink starter kit reagents (DUO92103, Sigma) according to manufacturer's instructions using antibodies against FLAG (goat, ab95045, Abcam) and NPM1 (mouse, ab10530, Abcam). After completion of the PLA protocol, we proceeded with incubation of a third antibody produced in rabbit to detect cellular compartments such as nucleolus with FBL (FIBRILLARIN, rabbit, ab184817 Abcam) or nuclear membrane with LMNB1 (LAMINB1, rabbit, ab133741, Abcam) following the procedure for immunofluorescence. Images were obtained with a Leica SP8 confocal microscope and processed with the LASX software.

### H3K79me2 ChIP-seq and bioinformatics analysis

N2a cells were transfected with shNPM1 (NPM1 KD) or shControl plasmid (CTR) and were grown on 6 wells plates (~ 6 Mio cells) for 72 h. Two independent biological replicates per condition was used. Each biological replicate corresponds to a different passage of N2a cells and for each biological replicate transfection was performed in a separate batch. N2a cells were fixed with freshly prepared room temperature 1% PFA (Life Technologies) for 5 min at room temperature. Fixation was stopped with 125 mM glycine, and fixed cells were washed 2 times with ice-cold PBS. Cells were collected in ice-cold PBS and centrifuged 5 min at 500 rpm. Lysis Buffer (1% SDS, 10 mM EDTA, 50 mM Tris HCl pH 8.0, Protease Inhibitor cocktail) was added to the pellet and incubated on ice for 5 min. Shearing was done using Bioruptor with the settings: 3 × 10 min 30 s pulse, 30 s pause and chromatin was centrifuged for 5 min at 13.000 rpm and the chromatin samples were saved. Chromatin samples were precleared with protein G dynabeads (Invitrogen). Genomic DNA regions of interest were isolated using 4 μg of H3K79me2 antibody (rabbit, ab3594, Abcam). Complexes were washed, eluted from the beads with SDS Buffer (1% SDS, 0.1 M NaHCO3), and subjected to RNase and Proteinase K treatment. Crosslink was reversed by incubation overnight at 65 °C, and ChIP DNA was purified by Qiagen MinElute kit according to the manufacturer’s instructions. Quantification was performed by using PicoDrop and the PicoGreen quantification kit (Lumiprobe). Libraries were prepared using the NEBNext Ultra II DNA library preparation kit for Illumina (Biolabs) and sequenced using HiSeq 3000 (Illumina) (paired-end, 51 bp reads). Galaxy platform was used for quality control, mapping, peak calling and differential enrichment analyses [1]. Mapping of reads was performed on mouse genome build mm10 (GRCm38) using Bowtie2 (v2.3.4.1; [36]. High quality and uniquely mapping reads were retained (mapq > 5). MACS2 callpeak (Galaxy Version 2.1.1.20160309.6; [79]) was used for peak calling using default parameters. Only the common peaks in both replicates were retained to prevent false-positive peaks in downstream analysis. Diffbind [55] was used for differential peak enrichment analysis (NPM1 KD/Control) using the default parameters on Galaxy (Galaxy Version 2.10.0). The input of the analysis are peaks datasets and the aligned reads for each replicate in each condition, as well as the input controls. The package normalises, models the data, and performs differential binding analysis between the conditions specified (similar to DESeq2). Thus, all aligned regions are considered for the final calculation of fold changes and significance. Coverage was computed using multiBamSummary, and.bam files were normalized by bamcompare and bigwigcompare (deeptools, Galaxy Version 3.3.2.0.0; [53]. All metaprofiles and heatmaps of ChIP-seq signals were generated with deeptools (Galaxy Version 3.3.2.0.0. Peaks were annotated and visualized using ChIPSeeker (Galaxy Version 1.18.0; (T. [70, 71]. GO-term enrichment analysis was performed using clusterProfiler (R, v. 3.10.1; (T. [70, 71] (Yu, Wang and He, 2015)).

### ChIP-qPCR

N2a cells were transfected with pVB-DOT1L-FLAG (Vector Builder) or pLKO.1-shCTR (Sigma, Non Target #3), pLKO.1-shNPM1 (Sigma, TRCN0000115430) as indicated and selected as described before. Chromatin was prepared as described for the H3K79me2 ChIP-seq, except for DOT1L ChIP by FLAG antibody. In this case, cells were treated with 1.5 mM EGS (ethylene glycol bis(succinimidyl succinate) for 30 min at RT before fixation with 1% PFA (Life Technologies) for 15 min at RT. The following antibody were used for the IP: H3K27me3 (rabbit, C15410195, diagenode); H3K79me2 (rabbit, ab3594, Abcam); FLAG (rabbit, 2368 s, cell signaling). Primers used for ChIP-qPCR are listed in Additional file 3: Table S2.

### RT-qPCR

Total RNA was extracted using the RNeasy kit (Qiagen). Reverse transcription was performed using the RevertAid RT Reverse Transcription kit (ThermoScientific) following the user´s manual instructions. Primers used for RT-qPCR are listed in Additional file 3: Table S2.

### Flow cytometry analysis of cell cycle

N2a cells (0.5–1 × 10^6^ cells) were harvested after transfection and washed with PBS. Cells were resuspended in 0.5 ml of PBS and fixed by adding the cell suspensions drop by drop to 4.5 ml 70% ethanol and stored overnight at 4 °C. The fixed cells were centrifuged at 200 g 5 min RT and washed with PBS three times. Cells were then resuspended in 1 ml of freshly prepared PI/Triton X-100 staining solution with RNase A (0.1% Triton X-100, 0.2 mg/ml DNase-free RNase in PBS and 200 µl of 1 mg/ml PI (Molecular Probes)) and incubated for 3 h at RT before being analyzed by flow cytometry.

### Supplementary Information

**Additional file 1: Figure S**1. **A) **Immunoblot analysis DOT1L/NPM1 interaction in N2a cell extract using either DOT1L antibody (DOT1L-IP) or NPM1 antibody (NPM1-IP). The asterisk indicates the position of endogenous DOT1L. **B) top)** single fluorescence channels corresponding to Fig. 1B; **bottom)** controls for proximity ligation assay (in situ PLA), showing stainings without both primary antibodies (left upper two panels), omitting one primary antibody at a time (NPM1, upper right two panels; FLAG, lower left two panels) or in presence of both primary antibodies (lower right two panels). In all cases, HA was used as DOT1L transfection control. (Scale bar 10µm). **C) top) **immunoblot analysis of DOT1L co-IP in control conditions or after EGS protein crosslinking using NPM1 antibody.We were not able to detect DOT1L in EGS conditions, probably due to the large size of protein complexes formed that are not able to enter the gel. The lower arrow indicates monomeric NPM1. The upper arrow indicates oligomeric NPM1 and in complex with additional proteins. Dash lines indicate the 55kDa (monomeric NPM1) and 130kDa (oligomeric NPM1) protein marker bands; **bottom)** the same membrane as in the top panel, left side, but probing was done against DOT1L using HA antibody. **Figure S2 A)** Picture of N2a cells growing in DMEM medium 3 days after NPM1 KD. Cells depleted of NPM1 (KD) consume less culture medium (pink medium) compared to control cells (yellow medium). **B)** Immunofluorescence image showing an example of N2a nuclei after 3 days of NPM1 knockdown (NPM1 KD) using GFP antibody to mark the transfected cells (red) and activated caspase 3 antibody (aCASP3, green) to mark cells undergoing apoptosis. Arrow indicates a cell undergoing nuclear fragmentation (NF) as shown by the lack of DAPI staining. (Scale bar 10µm). Images were taken using an AxioImager M2 fluorescence microscope with a 40x objective. **C)** Data point plot showing the percentage of transfected cells (GFP positive) expressing aCASP3 or undergoing nuclear fragmentation (NF) after 3 days of NPM1 knockdown. Statistical analysis was performed on n=3 biological replicates per condition on a total of n=176 (CTR), n=52 (KD) cells using an unpaired two tailed t-test; *p < 0.05, **p < 0.01. Error bars represent S.D. **D)** Flow cytometer analysis evaluating the cell cycle progression of control (CTR) and after 3 days of NPM1 knockdown (NPM1 KD) in N2a cells (left two panels). Stacked bar graphs showing the percentage of cells in different phases of the cell cycle in both conditions (CTR, NPM1 KD) (right panel). Statistical analysis was performed on n=3 biological replicates using a two-way ANOVA with Sidak’s post-hoc test. *p < 0.05, **p < 0.01. No significant differences were observed. **Figure S3**. A) Immunoblot of the two biological replicates used for the H3K79me2 ChIP-seq showing reduction after 3 days of NPM1 (KD) and concomitant increase of H3K79me2. GAPDH immunoblot was used as the normalization control.. B) Venn diagram depicting the intersection of H3K79me2 peaks in control (CTR) and after 3 days of NPM1 knockdown (NPM1 KD) in N2a cells.. C) Heatmap of H3K79me2 enrichment at the indicated genes (top) in control (CTR), after 3 days of NPM1 knockdown (NPM1 KD), and NPM1 KD/CTR conditions clustered into regions found 1 Kb up-/down-stream of scaled regions. Data is normalized by sequencing depth and input control (CTR/Input, NPM1 KD/Input, NPM1 KD/CTR). The metaprofiles show the log2(Fold Change) (LFC) of each gene. The scale corresponds to the log2(ChIP/Input) for control and NPM1 KD and to log2(ChIP NPM1 KD/ChIP CTR) for NPM1 KD/CTR.. D), E) Modified Integrative Genome Viewer (IGV) snapshot depicting the normalized H3K79me2 levels (Log2(H3K79me2 ChIP/Input)) at Ezh1 and Kdm3a genes in control (dark blue) or upon 3 days of NPM1 KD (light blue) cells, and the H3K79me2 signal ratio between NPM1 depletion and control (NPM1 KD/CTR, brown). The exact positions of the genes within the mouse mm10 genome and of the primers used for ChIP-qPCR are indicated at the bottom. Coverage is auto-scaled to fit the window. **Figure S4**. A) Modified Integrative Genome Viewer (IGV) snapshot depicting the normalized H3K79me2 levels (Log2(H3K79me2 ChIP/Input)) at Ctcf gene in control (dark blue) or upon 3 days of NPM1 KD (light blue), and the H3K79me2 signal ratio between NPM1 depletion and control (NPM1 KD/CTR, brown). The exact positions of the genes within the mm10 genome and of the primers used for ChIP-qPCR are indicated at the bottom. Coverage is auto-scaled to fit the window. B) Individual data points plot showing the fold change (NPM1 KD/CTR) expression of the indicated genes calculated after RT-qPCR upon 3 days of NPM1 KD in N2a cells. Gapdh was used as reference gene for normalization. Statistical analysis was performed on n=9 biological replicates using an unpaired two tailed Student's t-test; *p < 0.05, **p < 0.01. Outliers were identified using a Grubbs method. Error bars represent S.D. C) Line chart showing the dose dependent expression of Ezh1 (red lines) and Ezh2 (blue lines) to high Dot1l levels, or moderately increased Dot1l levels in black lines upon 3 days of NPM1 KD compared to controls, calculated after RT-qPCR. D) Immunoblot analysis of the levels of H3K79me2 upon 3 days of NPM1 KD in N2a cells. Ponceau staining was used for total protein normalization. E) Quantification of the signal intensities of the immunoblots shown in D, and in Fig. 4B, using Fiji (ImageJ). Protein levels were first normalized to the corresponding Ponceau signal and then presented as a ratio over the control values. Statistical analysis was performed on n=3 biological samples using an unpaired two tailed t-test. *p < 0.05, **p < 0.01. Error bars represent S.D. F) Individual data points plot showing the fold change expression (FC) of the indicated genes after RT-qPCR upon 3 days of NPM1 KD (blue) or DOT1L OE (red) in N2a cells. Gapdh was used as reference gene for normalization. Statistical analysis was performed on n=4 biological replicates using an unpaired one tailed (Npm1 and Dot1l) and two tailed t-test (all other genes). *p < 0.05, **p < 0.01. Outliers were identified using a Grubbs method. Error bars represent S.D. **Figure S5**. A) Immunoblot analysis of the levels of the H3K9ac, H3K9me2, H3K79me2 and NPM1 upon 3 days of NPM1 KD in N2a cells. Ponceau staining was used for total protein normalization. B) Quantification of the signal intensities of H3K9me2 and H3K9ac shown in A was done using Fiji (ImageJ). Protein levels were first normalized to the corresponding Ponceau signal and then presented as a ratio over the control values. Statistical analysis was performed on n=4 biological samples using an unpaired two tailed t-test. *p < 0.05, **p < 0.01. Error bars represent S.D. C) Individual data points plot showing the fold change enrichment over control of H3K79me2 calculated after ChIP-qPCR upon 3 days of NPM1 KD (KD/CTR). Abbreviations are: gSAT (gamma satellites), mSAT (major satellites). Statistical analysis was performed on n=4 biological replicates using an unpaired two tailed t-test. *p < 0.05, **p < 0.01. Error bars represent S.D. **Figure S6** A) Confocal immunofluorescence of N2a nuclei after 3 days of NPM1 KD and treatment with either EPZ or DMSO as control using FBL (FIBRILLARIN) antibody to highlight nucleolar shape. Note that CTR cells have larger and fewer nucleolar structures compared to NPM1 KD. EPZ treatment reverts only partially this phenotype, although the change is not statistically significant (Scale bars 20μm and 10μm). B) Quantification of the mean number (left) and area (right) of FBL (FIBRILLARIN) nucleolar stained structures shown in A. Statistical analysis was performed on n=2 biological replicates andthe following total number of nuclei: n=41 (CTR), n=35 (KD), n=17 (KD+EPZ) and n=35 (CTR+EPZ) to measure the number of nucleoli per nuclei , and n=24 (CTR), n=32 (KD), n=24 (KD+EPZ) and n=37 (CTR+EPZ) to measure the area per each condition using a two-way ANOVA with Sidak’s posthoc test. *p < 0.05, **p < 0.01. Error bars represent S.D. C) Confocal immunofluorescence of N2a nuclei after 3 days of NPM1 KD and treatment with either EPZ or DMSO as control using H3K79me2 antibody. Note that KD cells have increased H3K79me2 levels. EPZ treatment decreases H3K79me2 staining intensity. (Scale bars 50μm and 10μm). **Figure S7** A) and B) Modified Integrative Genome Viewer (IGV) snapshots depicting the normalized H3K79me2 levels (Log2(H3K79me2 ChIP/Input)) at Npm1 and Dot1l genes in control (dark blue) or upon 3 days of NPM1 KD (light blue), and the H3K79me2 signal ratio between NPM1 depletion and control (NPM1 KD/Control, brown). The exact positions of the genes within the mm10 genome and of the primers used for ChIP-qPCR are indicated at the bottom. Coverage is auto-scaled to fit the window. C) Representative immunoblot analysis of the levels of NPM1 and H3K79me2 upon 3 days of NPM1 KD in N2a cells. Ponceau staining was used for total protein normalization. D) Quantification of the signal intensities shown in C was done using Fiji (ImageJ). Protein levels were first normalized to the corresponding Ponceau signal and then presented as a ratio over the control values. Statistical analysis was performed on n=5 biological samples using an unpaired two tailed t-test. *p < 0.05, **p < 0.01. Error bars represent S.D. E) Confocal immunofluorescence of N2a cells showing NPM1 distribution in control transfected cells (GFP) or cells after 3 days of overexpressing DOT1L (DOT1L OE). (Scale bar 10µm). F) Confocal immunofluorescence of N2a cells showing NPM1 localization in DMSO treated cells (CTR) or cells treated for 48 hours with the DOT1L inhibitor EPZ. (Scale bar 10µm). **Figure S8** A) Immunoblot analysis confirming the overexpression of DOT1L (FLAG) after 3 days in N2a cells. Ponceau was used for total protein normalization. Note that the levels of H3K79me2 are also increasing.

**Additional file 2: Table S1**. List of genes with differential enrichment of H3K79me2 upon NPM1 KD.

**Additional file 3: Table S2**. List of primers used.

## Data Availability

Raw and normalized data generated in this work were deposited to GEO using the following accession number: GSE223363.
