# Supplementary material for: Nucleophosmin 1 cooperates with the methyltransferase DOT1L to preserve peri-nucleolar heterochromatin organization by regulating H3K27me3 levels and DNA repeats expression
Source: Epigenetics Chromatin. 2023 Sep 28;16:36. doi: 10.1186/s13072-023-00511-9 (PMC10537513; doi:10.1186/s13072-023-00511-9)

Additional Figure 1

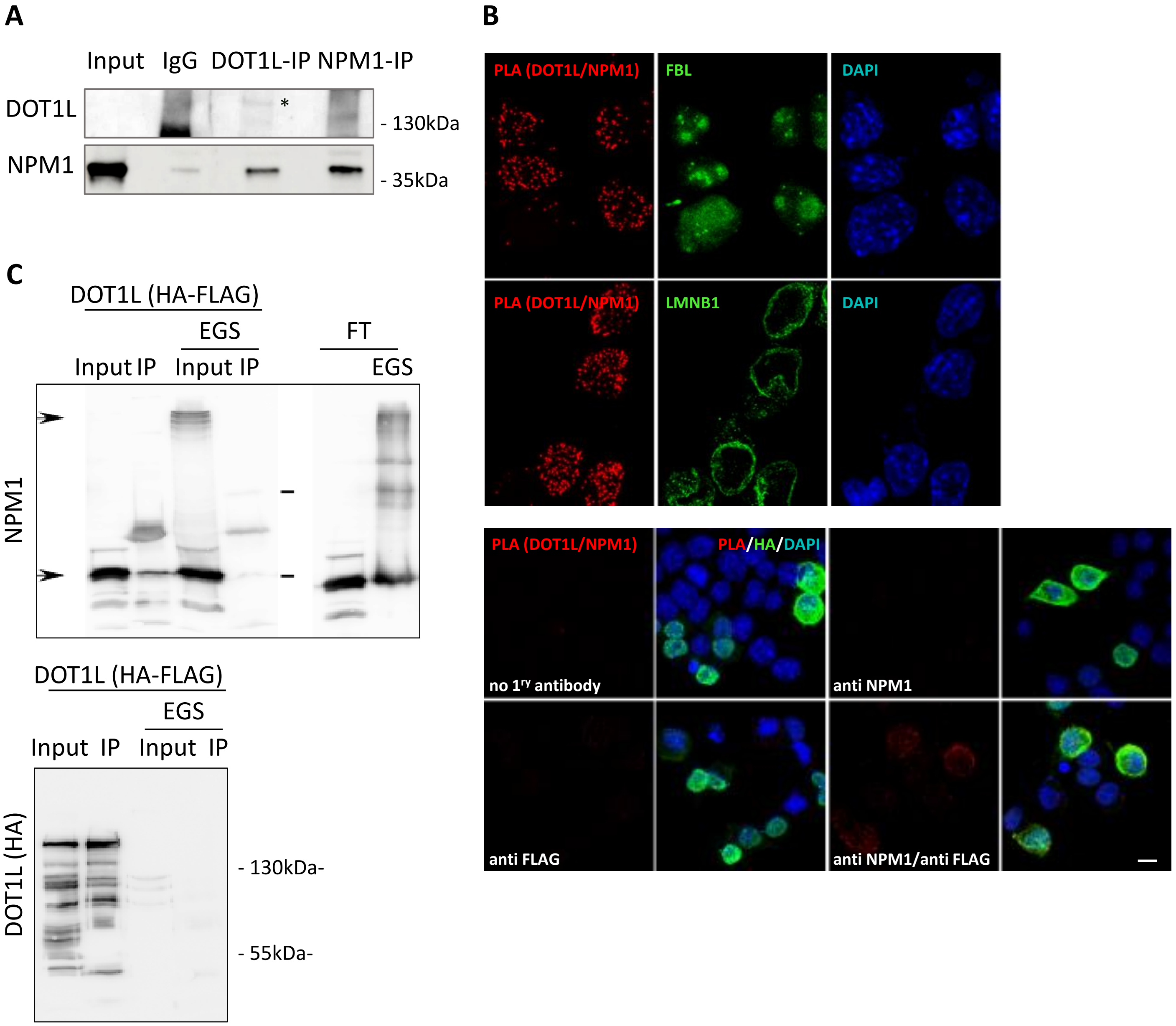

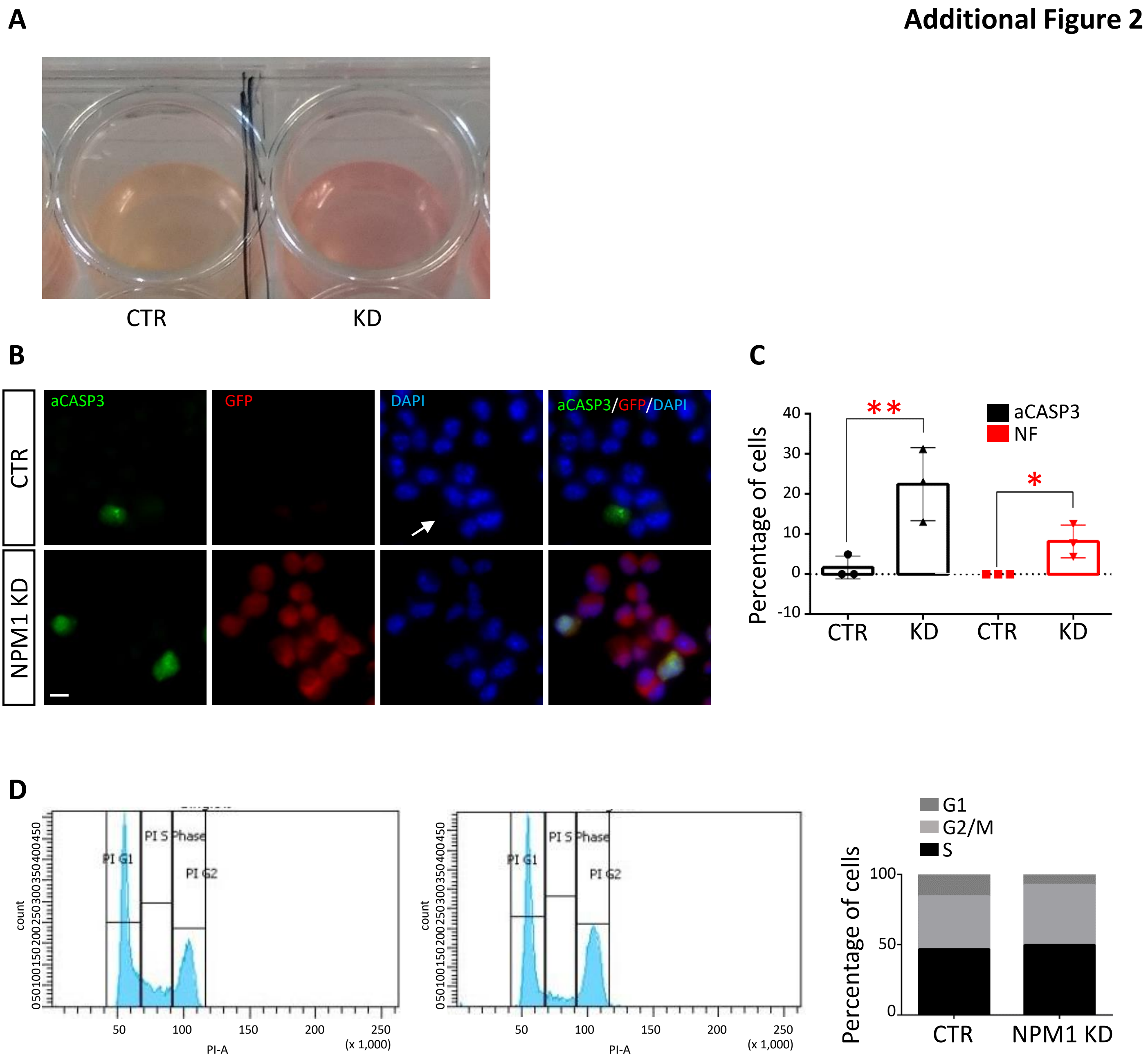
Additional Figure 2
A
CTR
KD
B
aCASP3
GFP
DAPI
aCASP3/GFP/DAPI
CTR
NPM1 KD
C
Percentage of cells
40
30
20
10
0
-10
**
*
aCASP3
NF
CTR
KD
CTR
KD
D
PI G1
PI S Phase
PI G2
count
PI-A
(x 1,000)
50
100
150
200
250
G1
G2/M
S
Percentage of cells
100
50
0
CTR
NPM1 KD

Additional Figure 3

A

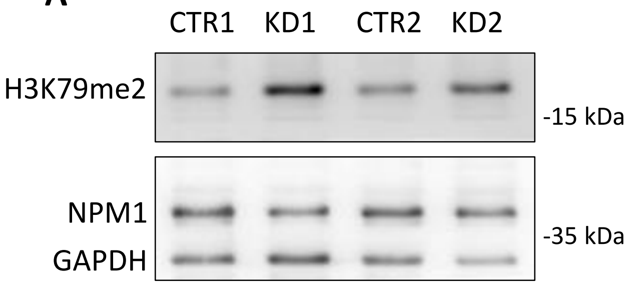

B

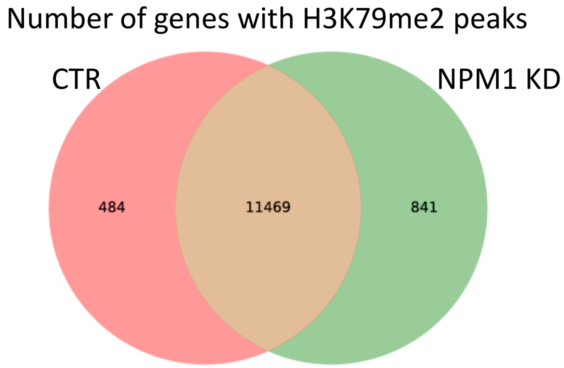

C

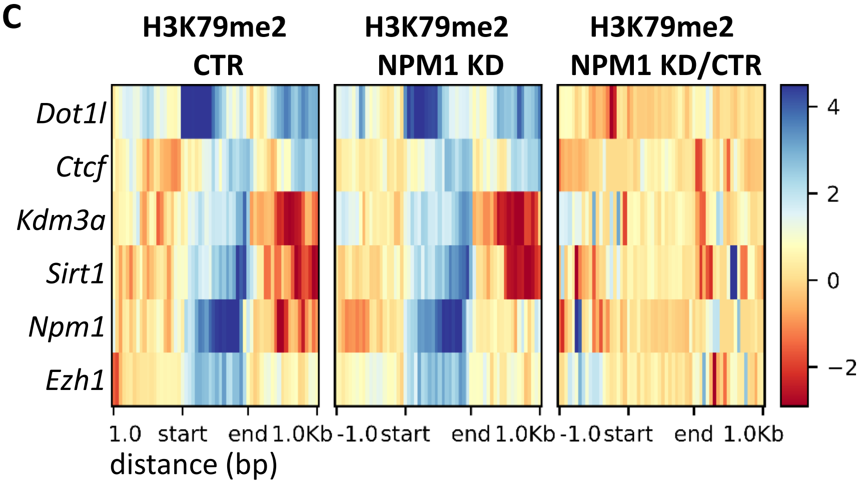

symbol	LFC	p.value	FDR	chrom	start	end
Dot1l	-0.21	0.00118	0.0186	chr10	80755027	80774628
Ctcf	0.35	0.00025	0.00557	chr8	105622303	105636382
Kdm3a	0.35	0.000152	0.00384	chr6	71612848	71632976
Sirt1	0.29	2.74e-05	0.00102	chr10	63326541	63338358
Npm1	-0.18	0.000598	0.0111	chr11	33150860	33164662
Ezh1	0.27	0.00135	0.0207	chr11	101218122	101226955

D

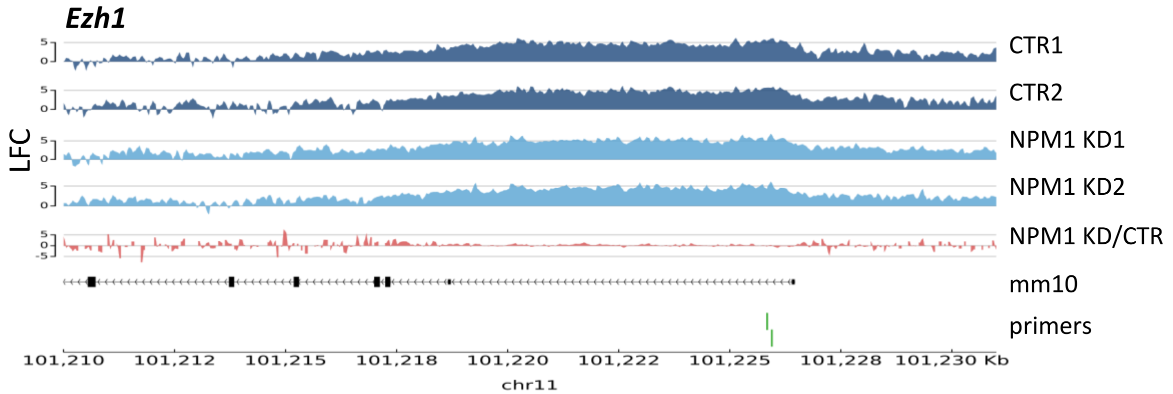

E

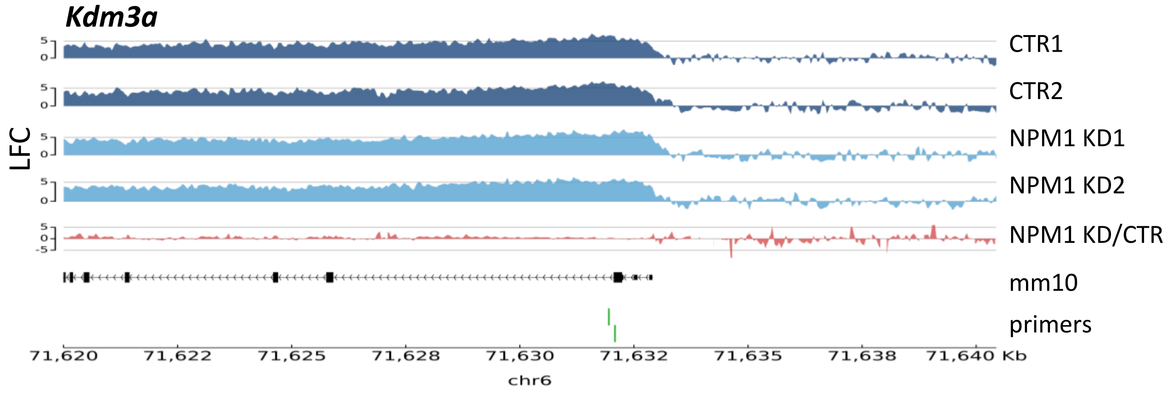

Additional Figure 4

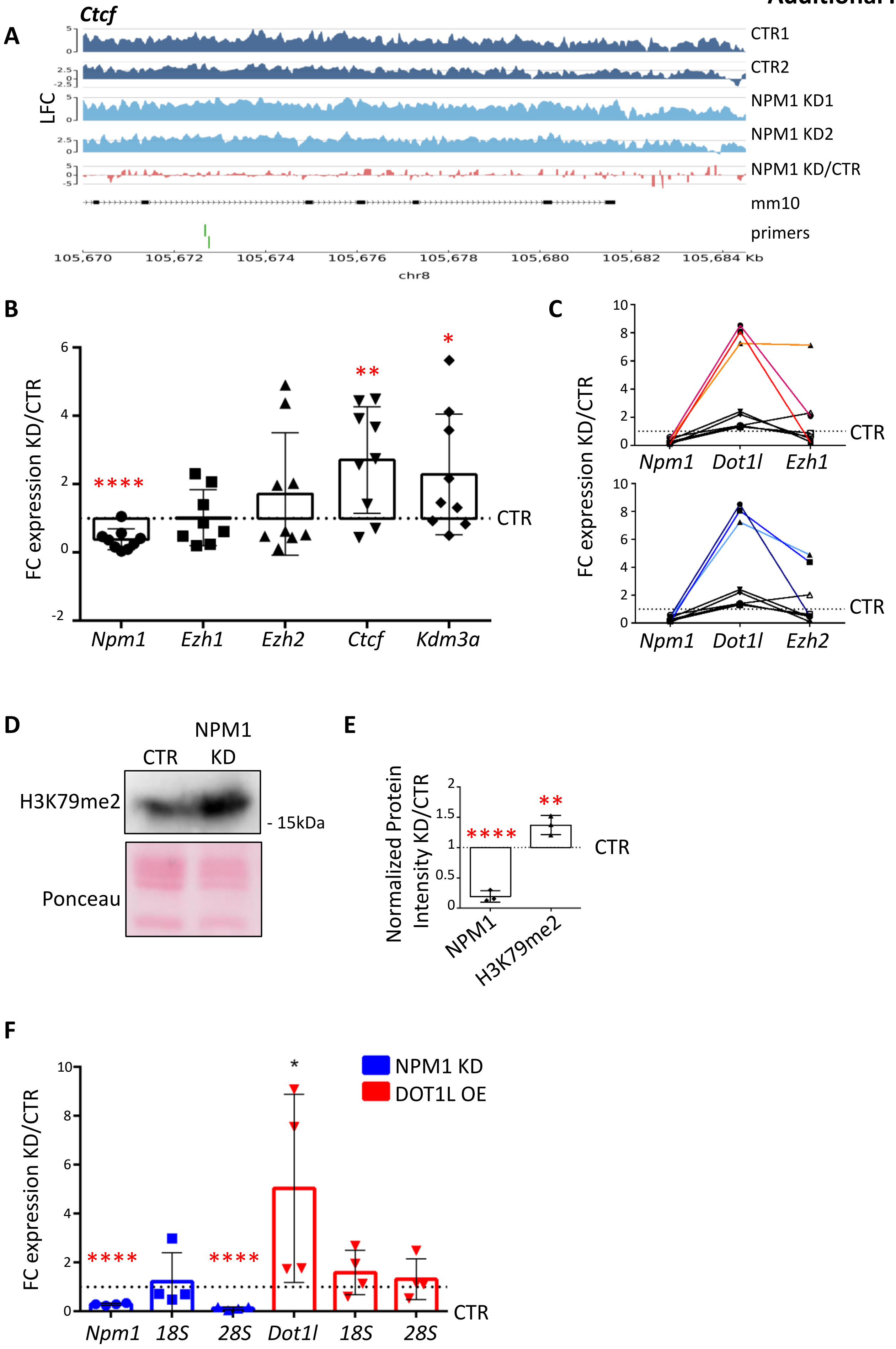

Additional Figure 5

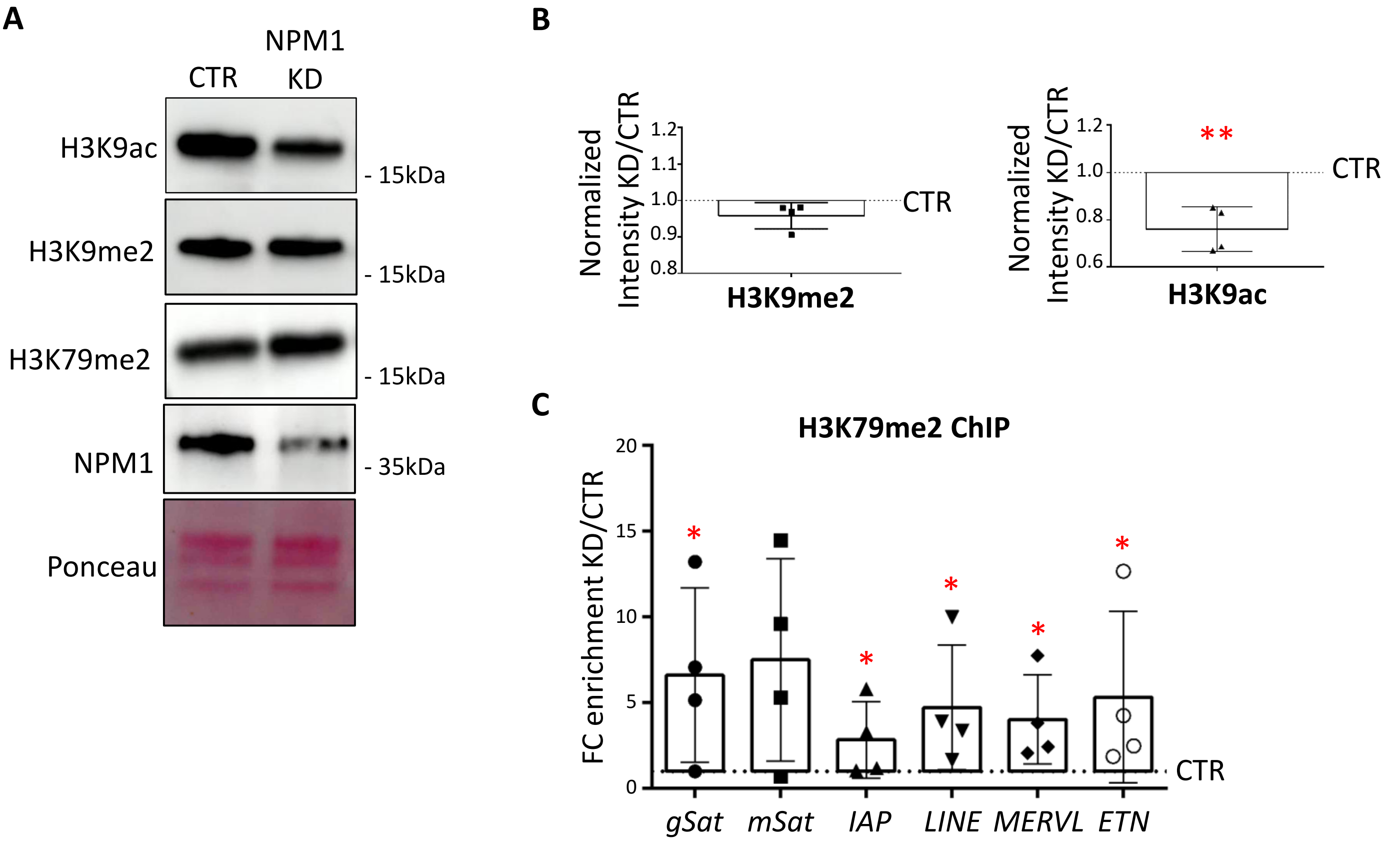

Additional Figure 6

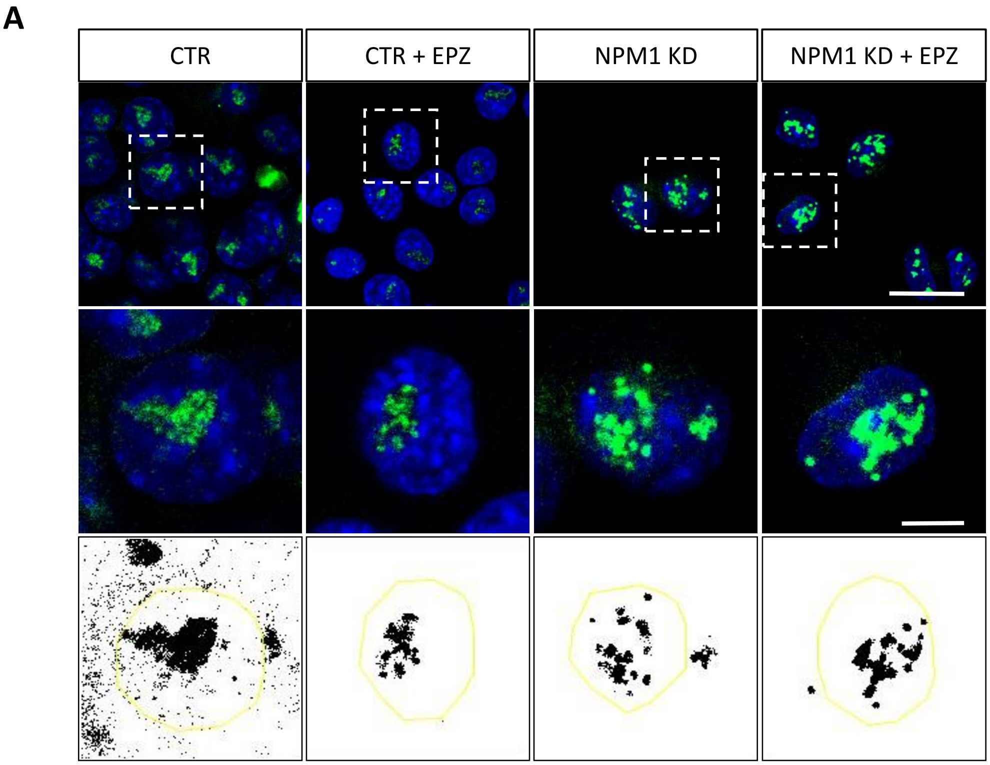

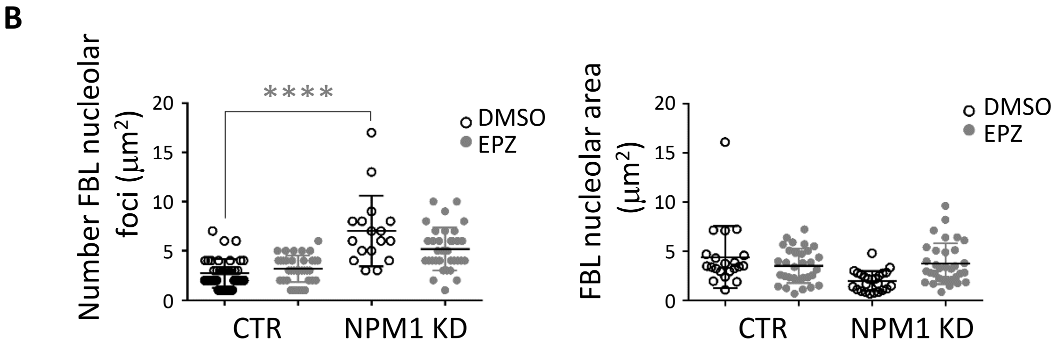

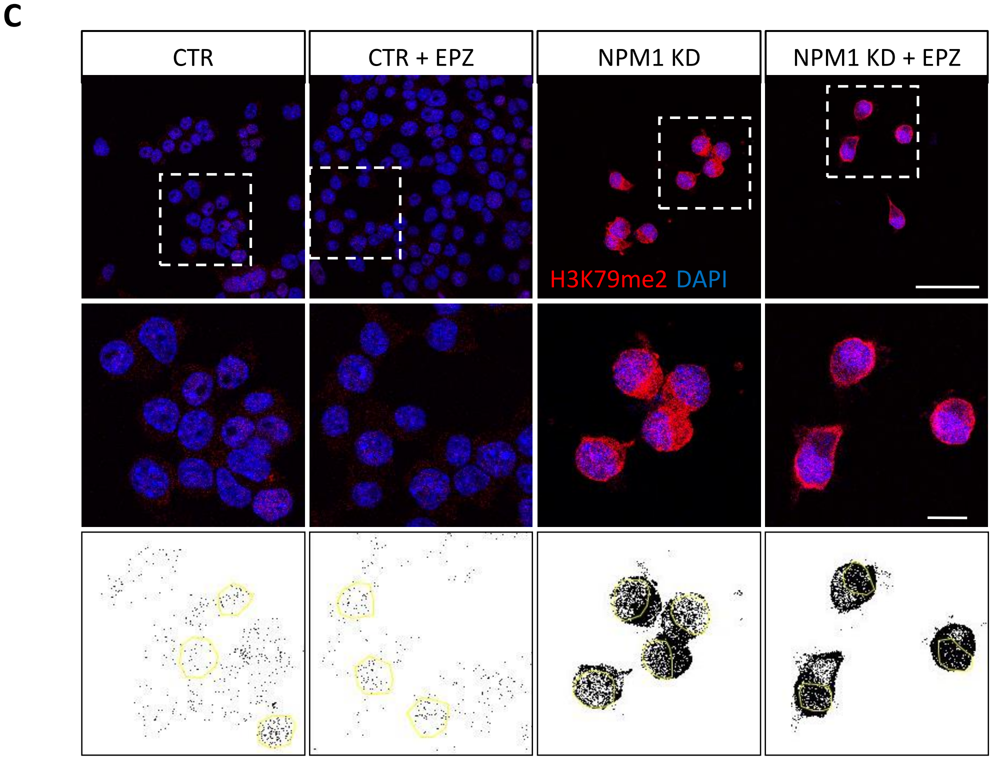

Additional Figure 7

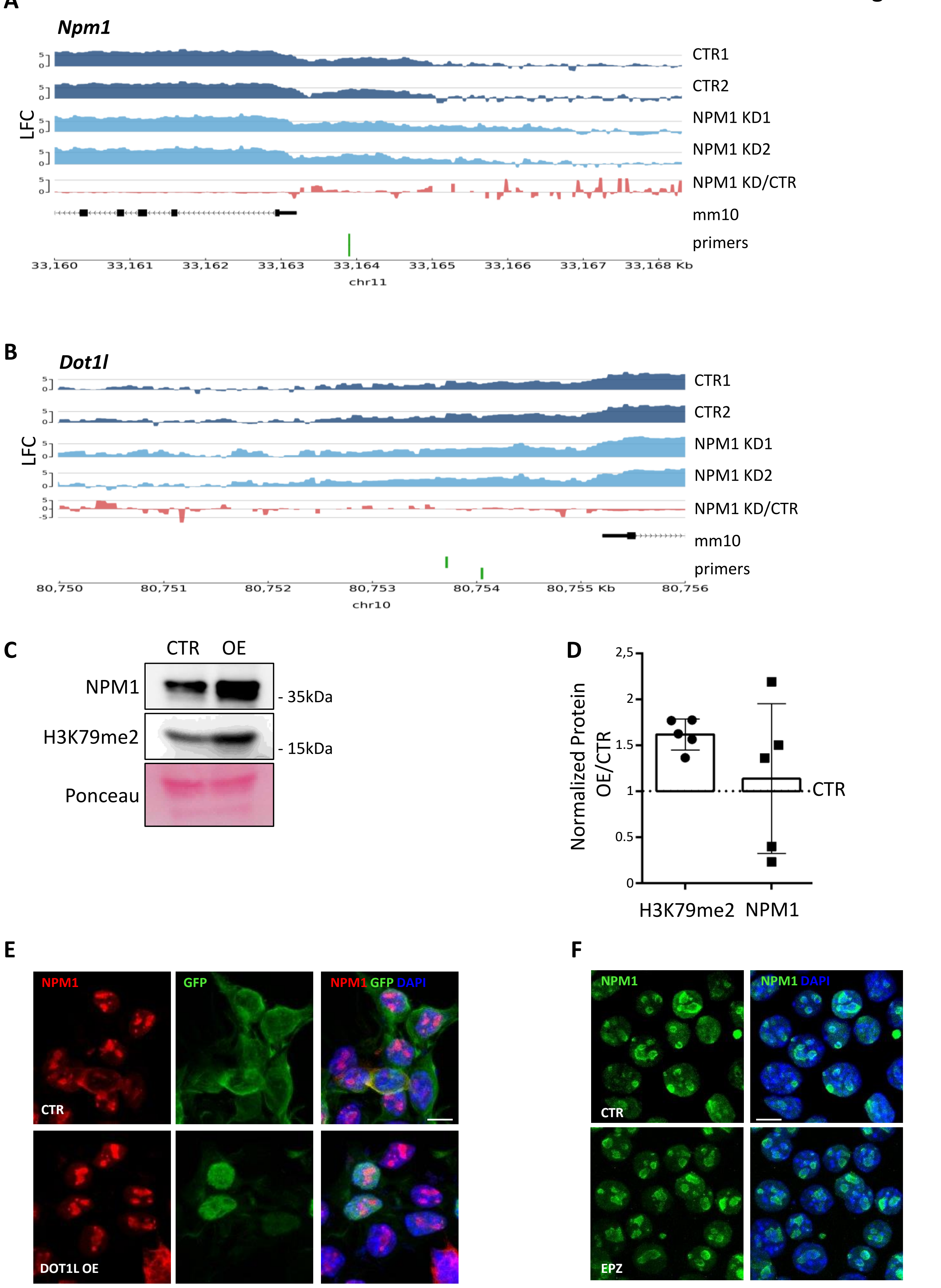

Additional Figure 8

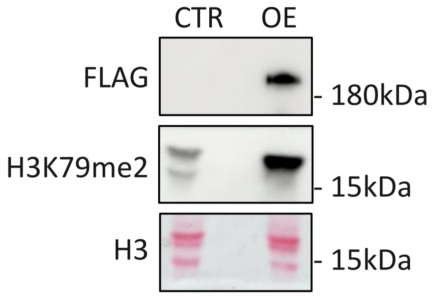

Supplement: Supplementary file 1 — Additional file 1: Figure S1. A) Immunoblot analysis DOT1L/NPM1 interaction in N2a cell extract using either DOT1L antibody (DOT1L-IP) or NPM1 antibody (NPM1-IP). The asterisk indicates the position of endogenous DOT1L. B) top) single fluorescence channels corresponding to Fig. 1B; bottom) controls for proximity ligation assay (in situ PLA), showing stainings without both primary antibodies (left upper two panels), omitting one primary antibody at a time (NPM1, upper right two panels; FLAG, lower left two panels) or in presence of both primary antibodies (lower right two panels). In all cases, HA was used as DOT1L transfection control. (Scale bar 10µm). C) top) immunoblot analysis of DOT1L co-IP in control conditions or after EGS protein crosslinking using NPM1 antibody.We were not able to detect DOT1L in EGS conditions, probably due to the large size of protein complexes formed that are not able to enter the gel. The lower arrow indicates monomeric NPM1. The upper arrow indicates oligomeric NPM1 and in complex with additional proteins. Dash lines indicate the 55kDa (monomeric NPM1) and 130kDa (oligomeric NPM1) protein marker bands; bottom) the same membrane as in the top panel, left side, but probing was done against DOT1L using HA antibody. Figure S2 A) Picture of N2a cells growing in DMEM medium 3 days after NPM1 KD. Cells depleted of NPM1 (KD) consume less culture medium (pink medium) compared to control cells (yellow medium). B) Immunofluorescence image showing an example of N2a nuclei after 3 days of NPM1 knockdown (NPM1 KD) using GFP antibody to mark the transfected cells (red) and activated caspase 3 antibody (aCASP3, green) to mark cells undergoing apoptosis. Arrow indicates a cell undergoing nuclear fragmentation (NF) as shown by the lack of DAPI staining. (Scale bar 10µm). Images were taken using an AxioImager M2 fluorescence microscope with a 40x objective. C) Data point plot showing the percentage of transfected cells (GFP positive) expressing aCASP3 or [file 13072_2023_511_MOESM1_ESM.zip › New folder/E&C_Additional_Files_Izzo.pdf]
