# Supplementary material for: Nucleophosmin 1 cooperates with the methyltransferase DOT1L to preserve peri-nucleolar heterochromatin organization by regulating H3K27me3 levels and DNA repeats expression
Source: Epigenetics Chromatin. 2023 Sep 28;16:36. doi: 10.1186/s13072-023-00511-9 (PMC10537513; doi:10.1186/s13072-023-00511-9)
Supplement: Supplementary file 1 — Additional file 1: Figure S1. A) Immunoblot analysis DOT1L/NPM1 interaction in N2a cell extract using either DOT1L antibody (DOT1L-IP) or NPM1 antibody (NPM1-IP). The asterisk indicates the position of endogenous DOT1L. B) top) single fluorescence channels corresponding to Fig. 1B; bottom) controls for proximity ligation assay (in situ PLA), showing stainings without both primary antibodies (left upper two panels), omitting one primary antibody at a time (NPM1, upper right two panels; FLAG, lower left two panels) or in presence of both primary antibodies (lower right two panels). In all cases, HA was used as DOT1L transfection control. (Scale bar 10µm). C) top) immunoblot analysis of DOT1L co-IP in control conditions or after EGS protein crosslinking using NPM1 antibody.We were not able to detect DOT1L in EGS conditions, probably due to the large size of protein complexes formed that are not able to enter the gel. The lower arrow indicates monomeric NPM1. The upper arrow indicates oligomeric NPM1 and in complex with additional proteins. Dash lines indicate the 55kDa (monomeric NPM1) and 130kDa (oligomeric NPM1) protein marker bands; bottom) the same membrane as in the top panel, left side, but probing was done against DOT1L using HA antibody. Figure S2 A) Picture of N2a cells growing in DMEM medium 3 days after NPM1 KD. Cells depleted of NPM1 (KD) consume less culture medium (pink medium) compared to control cells (yellow medium). B) Immunofluorescence image showing an example of N2a nuclei after 3 days of NPM1 knockdown (NPM1 KD) using GFP antibody to mark the transfected cells (red) and activated caspase 3 antibody (aCASP3, green) to mark cells undergoing apoptosis. Arrow indicates a cell undergoing nuclear fragmentation (NF) as shown by the lack of DAPI staining. (Scale bar 10µm). Images were taken using an AxioImager M2 fluorescence microscope with a 40x objective. C) Data point plot showing the percentage of transfected cells (GFP positive) expressing aCASP3 or [file 13072_2023_511_MOESM1_ESM.zip › New folder/E&C_Immunoblots.pdf]

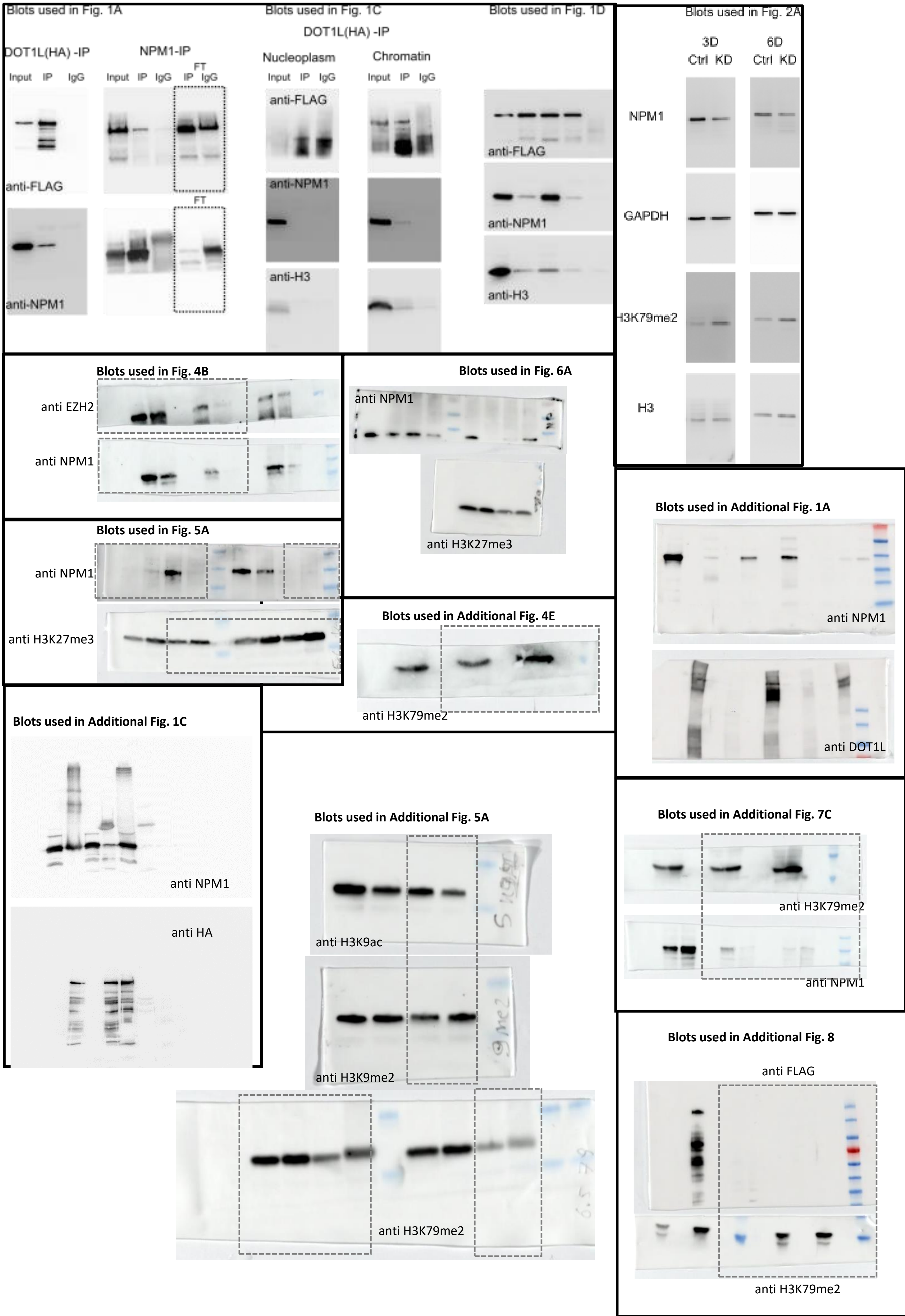

Blots used in Fig. 1A
DOT1L(HA) -IP
Input IP IgG
NPM1-IP
Input IP IgG
FT
IP IgG
anti-FLAG
FT
anti-NPM1
Blots used in Fig. 1C
DOT1L(HA) -IP
Nucleoplasm
Chromatin
Input IP IgG
Input IP IgG
anti-FLAG
anti-NPM1
anti-H3
Blots used in Fig. 1D
anti-FLAG
anti-NPM1
anti-H3
Blots used in Fig. 2A
3D
6D
Ctrl KD
Ctrl KD
NPM1
GAPDH
H3K79me2
H3
Blots used in Fig. 4B
anti EZH2
anti NPM1
Blots used in Fig. 6A
anti NPM1
anti H3K27me3
Blots used in Fig. 5A
anti NPM1
anti H3K27me3
Blots used in Additional Fig. 1A
anti NPM1
anti DOT1L
Blots used in Additional Fig. 4E
anti H3K79me2
Blots used in Additional Fig. 1C
anti NPM1
anti HA
Blots used in Additional Fig. 5A
anti H3K9ac
anti H3K9me2
anti H3K79me2
Blots used in Additional Fig. 7C
anti H3K79me2
anti NPM1
Blots used in Additional Fig. 8
anti FLAG
anti H3K79me2
