# Supplementary material for: Nucleophosmin 1 cooperates with the methyltransferase DOT1L to preserve peri-nucleolar heterochromatin organization by regulating H3K27me3 levels and DNA repeats expression
Source: Epigenetics Chromatin. 2023 Sep 28;16:36. doi: 10.1186/s13072-023-00511-9 (PMC10537513; doi:10.1186/s13072-023-00511-9)
Supplement: Supplementary file 3 — Additional file 3: Table S2. List of primers used. [file 13072_2023_511_MOESM3_ESM.docx]

**Additional Figures and Tables**

**Additional Figures**

**Additional Figure 1**

**A)** Immunoblot analysis DOT1L/NPM1 interaction in N2a cell extract using either DOT1L antibody (DOT1L-IP) or NPM1 antibody (NPM1-IP). The asterisk indicates the position of endogenous DOT1L.

**B) top)** single fluorescence channels corresponding to Fig. 1B; **bottom)** controls for proximity ligation assay (in situ PLA), showing stainings without both primary antibodies (left upper two panels), omitting one primary antibody at a time (NPM1, upper right two panels; FLAG, lower left two panels) or in presence of both primary antibodies (lower right two panels). In all cases, HA was used as DOT1L transfection control. (Scale bar 10µm).

**C) top)** immunoblot analysis of DOT1L co-IP in control conditions or after EGS protein crosslinking using NPM1 antibody.We were not able to detect DOT1L in EGS conditions, probably due to the large size of protein complexes formed that are not able to enter the gel. The lower arrow indicates monomeric NPM1. The upper arrow indicates oligomeric NPM1 and in complex with additional proteins. Dash lines indicate the 55kDa (monomeric NPM1) and 130kDa (oligomeric NPM1) protein marker bands; **bottom)** the same membrane as in the top panel, left side, but probing was done against DOT1L using HA antibody.

**Additional Figure 2**

**A)** Picture of N2a cells growing in DMEM medium 3 days after NPM1 KD. Cells depleted of NPM1 (KD) consume less culture medium (pink medium) compared to control cells (yellow medium).

**B)** Immunofluorescence image showing an example of N2a nuclei after 3 days of NPM1 knockdown (NPM1 KD) using GFP antibody to mark the transfected cells (red) and activated caspase 3 antibody (aCASP3, green) to mark cells undergoing apoptosis. Arrow indicates a cell undergoing nuclear fragmentation (NF) as shown by the lack of DAPI staining. (Scale bar 10µm). Images were taken using an AxioImager M2 fluorescence microscope with a 40x objective.

**C)** Data point plot showing the percentage of transfected cells (GFP positive) expressing aCASP3 or undergoing nuclear fragmentation (NF) after 3 days of NPM1 knockdown. Statistical analysis was performed on n=3 biological replicates per condition on a total of n=176 (CTR), n=52 (KD) cells using an unpaired two tailed t-test; *p < 0.05, **p < 0.01. Error bars represent S.D.

**D)** Flow cytometer analysis evaluating the cell cycle progression of control (CTR) and after 3 days of NPM1 knockdown (NPM1 KD) in N2a cells (left two panels). Stacked bar graphs showing the percentage of cells in different phases of the cell cycle in both conditions (CTR, NPM1 KD) (right panel). Statistical analysis was performed on n=3 biological replicates using a two-way ANOVA with Sidak’s post-hoc test. *p < 0.05, **p < 0.01. No significant differences were observed.

**Additional Figure 3**

**A)** Immunoblot of the two biological replicates used for the H3K79me2 ChIP-seq showing reduction after 3 days of NPM1 (KD) and concomitant increase of H3K79me2. GAPDH immunoblot was used as the normalization control.

**B)** Venn diagram depicting the intersection of H3K79me2 peaks in control (CTR) and after 3 days of NPM1 knockdown (NPM1 KD) in N2a cells.

**C)** Heatmap of H3K79me2 enrichment at the indicated genes (top) in control (CTR), after 3 days of NPM1 knockdown (NPM1 KD), and NPM1 KD/CTR conditions clustered into regions found 1 Kb up-/down-stream of scaled regions. Data is normalized by sequencing depth and input control (CTR/Input, NPM1 KD/Input, NPM1 KD/CTR). The metaprofiles show the log2(Fold Change) (LFC) of each gene. The scale corresponds to the log2(ChIP/Input) for control and NPM1 KD and to log2(ChIP NPM1 KD/ChIP CTR) for NPM1 KD/CTR.

**D), E)** Modified Integrative Genome Viewer (IGV) snapshot depicting the normalized H3K79me2 levels (Log2(H3K79me2 ChIP/Input)) at *Ezh1* and *Kdm3a* genes in control (dark blue) or upon 3 days of NPM1 KD (light blue) cells, and the H3K79me2 signal ratio between NPM1 depletion and control (NPM1 KD/CTR, brown). The exact positions of the genes within the mouse mm10 genome and of the primers used for ChIP-qPCR are indicated at the bottom. Coverage is auto-scaled to fit the window.

**Additional Figure 4**

**A)** Modified Integrative Genome Viewer (IGV) snapshot depicting the normalized H3K79me2 levels (Log2(H3K79me2 ChIP/Input)) at *Ctcf* gene in control (dark blue) or upon 3 days of NPM1 KD (light blue), and the H3K79me2 signal ratio between NPM1 depletion and control (NPM1 KD/CTR, brown). The exact positions of the genes within the mm10 genome and of the primers used for ChIP-qPCR are indicated at the bottom. Coverage is auto-scaled to fit the window.

**B)** Individual data points plot showing the fold change (NPM1 KD/CTR) expression of the indicated genes calculated after RT-qPCR upon 3 days of NPM1 KD in N2a cells. *Gapdh* was used as reference gene for normalization. Statistical analysis was performed on n=9 biological replicates using an unpaired two tailed Student's t-test; *p < 0.05, **p < 0.01. Outliers were identified using a Grubbs method. Error bars represent S.D.

**C)** Line chart showing the dose dependent expression of *Ezh1* (red lines) and *Ezh2* (blue lines) to high *Dot1l* levels, or moderately increased *Dot1l* levels in black lines upon 3 days of NPM1 KD compared to controls, calculated after RT-qPCR.

**D)** Immunoblot analysis of the levels of H3K79me2 upon 3 days of NPM1 KD in N2a cells. Ponceau staining was used for total protein normalization.

**E)** Quantification of the signal intensities of the immunoblots shown in D, and in Fig. 4B, using Fiji (ImageJ). Protein levels were first normalized to the corresponding Ponceau signal and then presented as a ratio over the control values. Statistical analysis was performed on n=3 biological samples using an unpaired two tailed t-test. *p < 0.05, **p < 0.01. Error bars represent S.D.

**F)** Individual data points plot showing the fold change expression (FC) of the indicated genes after RT-qPCR upon 3 days of NPM1 KD (blue) or DOT1L OE (red) in N2a cells. Gapdh was used as reference gene for normalization. Statistical analysis was performed on n=4 biological replicates using an unpaired one tailed (Npm1 and Dot1l) and two tailed t-test (all other genes). *p < 0.05, **p < 0.01. Outliers were identified using a Grubbs method. Error bars represent S.D.

**Additional Figure 5**

**A)** Immunoblot analysis of the levels of the H3K9ac, H3K9me2, H3K79me2 and NPM1 upon 3 days of NPM1 KD in N2a cells. Ponceau staining was used for total protein normalization.

**B)** Quantification of the signal intensities of H3K9me2 and H3K9ac shown in A was done using Fiji (ImageJ). Protein levels were first normalized to the corresponding Ponceau signal and then presented as a ratio over the control values. Statistical analysis was performed on n=4 biological samples using an unpaired two tailed t-test. *p < 0.05, **p < 0.01. Error bars represent S.D.

**C)** Individual data points plot showing the fold change enrichment over control of H3K79me2 calculated after ChIP-qPCR upon 3 days of NPM1 KD (KD/CTR). Abbreviations are: gSAT (gamma satellites), mSAT (major satellites). Statistical analysis was performed on n=4 biological replicates using an unpaired two tailed t-test. *p < 0.05, **p < 0.01. Error bars represent S.D.

**Additional Figure 6**

**A)** Confocal immunofluorescence of N2a nuclei after 3 days of NPM1 KD and treatment with either EPZ or DMSO as control using FBL (FIBRILLARIN) antibody to highlight nucleolar shape. Note that CTR cells have larger and fewer nucleolar structures compared to NPM1 KD. EPZ treatment reverts only partially this phenotype, although the change is not statistically significant (Scale bars 20μm and 10μm).

**B)** Quantification of the mean number (left) and area (right) of FBL (FIBRILLARIN) nucleolar stained structures shown in A. Statistical analysis was performed on n=2 biological replicates andthe following total number of nuclei: n=41 (CTR), n=35 (KD), n=17 (KD+EPZ) and n=35 (CTR+EPZ) to measure the number of nucleoli per nuclei , and n=24 (CTR), n=32 (KD), n=24 (KD+EPZ) and n=37 (CTR+EPZ) to measure the area per each condition using a two-way ANOVA with Sidak’s posthoc test. *p < 0.05, **p < 0.01. Error bars represent S.D.

**C)** Confocal immunofluorescence of N2a nuclei after 3 days of NPM1 KD and treatment with either EPZ or DMSO as control using H3K79me2 antibody. Note that KD cells have increased H3K79me2 levels. EPZ treatment decreases H3K79me2 staining intensity. (Scale bars 50μm and 10μm).

**Additional Figure 7**

**A)** and **B)** Modified Integrative Genome Viewer (IGV) snapshots depicting the normalized H3K79me2 levels (Log2(H3K79me2 ChIP/Input)) at *Npm1* and *Dot1l* genes in control (dark blue) or upon 3 days of NPM1 KD (light blue), and the H3K79me2 signal ratio between NPM1 depletion and control (NPM1 KD/Control, brown). The exact positions of the genes within the mm10 genome and of the primers used for ChIP-qPCR are indicated at the bottom. Coverage is auto-scaled to fit the window.

**C)** Representative immunoblot analysis of the levels of NPM1 and H3K79me2 upon 3 days of NPM1 KD in N2a cells. Ponceau staining was used for total protein normalization.

**D)** Quantification of the signal intensities shown in C was done using Fiji (ImageJ). Protein levels were first normalized to the corresponding Ponceau signal and then presented as a ratio over the control values. Statistical analysis was performed on n=5 biological samples using an unpaired two tailed t-test. *p < 0.05, **p < 0.01. Error bars represent S.D.

**E)** Confocal immunofluorescence of N2a cells showing NPM1 distribution in control transfected cells (GFP) or cells after 3 days of overexpressing DOT1L (DOT1L OE). (Scale bar 10µm).

**F)** Confocal immunofluorescence of N2a cells showing NPM1 localization in DMSO treated cells (CTR) or cells treated for 48 hours with the DOT1L inhibitor EPZ. (Scale bar 10µm).

**Additional Figure 8**

**A)** Immunoblot analysis confirming the overexpression of DOT1L (FLAG) after 3 days in N2a cells. Ponceau was used for total protein normalization. Note that the levels of H3K79me2 are also increasing.

**Additional Tables**

**Additional Table 1**

List of genes with differential enrichment of H3K79me2 upon NPM1 KD

**Additional Table 2**

List of primers used

| **Target** | **Purpose** | **Forward Sequence (5´- 3´)** |
| --- | --- | --- |
| gamma satellite (gSat) | ChIP/RT | F: GACGACTTGAAAAATGACGAAATC |
|  | ChIP/RT | R: CATATTCCAGGTCCTTCAGTGTGC |
| major satellite (mSat) | ChIP/RT | F: GACGACTTGAAAAATGACGAAATC |
|  | ChIP/RT | R: CATATTCCAGGTCCTTCAGTGTGC |
| IAP | ChIP/RT | F: AAGCAGCAATCACCCACTTTGG |
|  | ChIP/RT | R: CAATCATTAGATGTGGCTGCCAAG |
| LINE | ChIP/RT | F: GACATAGACTAACAAACTGGCTACACAAAC |
|  | ChIP/RT | R: GGTAGTGTCTATCTTTTTCTCTGAGATGAG |
| MERVL | ChIP/RT | F: TTCTTCTAGACCTGTAACCAGACTCA |
|  | ChIP/RT | R: TCCTTAGTAGTGTAGCGAATTTCCTC |
| ETN | ChIP/RT | F: GTGGTATCTCAGGAGGAGTGCC |
|  | ChIP/RT | R: GGGCAGCTCCTCTATCTGAGTG |
| *Dot1l* | RT | F: CGAGCCCGCCGTCTAC |
|  | RT | R: GTCTCAATAATCTCATGAGCAGC |
| *Npm1* | RT | F: CACCAGTTGTCATTAAGAACGGT |
|  | RT | R: GACTGCCTTCATAGTTCATTGCT |
| *Ezh1* | RT | F: TGAAATCTGAGTATATGCGGC |
|  | RT | R: AGATATCCTGGCTGTCGAAC |
| *Ezh2* | RT | F: GCCAGACTGGGAAGAAATCTG |
|  | RT | R: TGTGCTGGAAAATCCAAGTCA |
| *Ctcf* | RT | F: GTGGAGACACTAGAGCAGGG |
|  | RT | R: CTCTGCATTAACCTCAGACAGC |
| *Kdm3a* | RT | F: GCCTTCCAGGTTTGATGATCTG |
|  | RT | R: TGCCATCTCGCCTGGTGTA |
| *Gapdh* | RT | F: CGGCCGCATCTTCTTGTG |
|  | RT | R: TGACCAGGCGCCCAATAC |
| 18S | RT | F: AAACGGCTACCACATCCAAG |
|  | RT | R: CCTCCAATGGATCCTCGTTA |
| 28S | RT | F: CCCAGTGCTCTGAATGTCAA |
|  | RT | R: ATGACGAGGCATTTGGCTAC |
| *Dot1l* | ChIP | F: GGGGAAGCAAGCCGTAAAGA |
|  | ChIP | R: CAAGTTGGGGAGGAAAGGGTT |
| *Npm1* | ChIP | F: TGACAGCGGCCCTAATCTTTT |
|  | ChIP | R: TCTGACACGTCTCTGGAGTCT |
| *Ezh1* | ChIP | F: TTTCTGGGAATTCTGGGGACG |
|  | ChIP | R: CGGCACCTCCTCAACATTTTG |
| *Ezh2* | ChIP | F: TCACCCCATGTTTTCTCAGCA |
|  | ChIP | R: GGTTGGGCTGCGTACTGTATA |
| *Ctcf* | ChIP | F: CCCTCGGGTTGTTGtttgttt |
|  | ChIP | R: CTGAGATGGCCTGGGTTCAAT |
| *Kdm3a* | ChIP | F: TCAACTGTCCCGTTACCAAGG |
|  | ChIP | R: CTGTTTCCCACACCGACGTTA |
| C3T2.1 | ChIP | F: TTCTTCTGGGCGCTCTCTCG |
|  | ChIP | R:GAAGGGGAACATCAGAGACCAC |
| MSR 3 | FISH | /5Cy3/A+AT+GA+GA+AA+CA+TC+CA+CT+TG+AC+GA+CT |
| MSR 4 | FISH | /5Cy3/T+GA+AA+AA+TG+AG+AA+AT+GC+AC+AC+TG |
